# Asia Pacific Conference on Vision 2016 Abstracts

**DOI:** 10.1177/2041669516685789

**Published:** 2016-12-01

The 12th annual Asia Pacific Conference on Vision was held in Fremantle, Western Australia. There were 152 researchers from 13 different countries who presented work on a broad range of Vision Science in a very congenial atmosphere. The Organising Committee (Chair: David Badcock, Jason Bell, Kate Crookes, Romina Palermo and Troy Visser; all from the University of Western Australia) and Robert O’Shea (Murdoch University) thank them all for their contributions.

The Abstracts are provided below. Keynote talks are presented first and then the others are listed in order of presentation.

## Keynotes

## Motion Perception: From a Dark Room to the Real World

**Shin’ya Nishida**

NTT Communication Science Laboratories, Atsugi, Japan

Many researchers, including me, have been investigating basic computations of visual motion processing (e.g., motion detection, motion integration, local speed and direction estimation, optic flow processing, cross-attribute interactions) using a variety of specialized stimuli for lab experiments (e.g., drifting gratings, random-dot kinematograms, plaids, drift-balanced second-order motion). In the first half of my presentation, I will briefly introduce some of my studies along this line, including those using Global Gabor motion stimuli that revealed interactions among several stages of visual motion processing (local motion detection, motion integration and optic flow processing). In the latter half, I will introduce a couple of our recent studies addressing motion perception in the real world. One is about how we use motion information to see material properties. We found that characteristic structures of motion flow and image deformation are useful information for us to see opaque and transparent liquids and their viscosity. The other study, which is more application oriented, is a projection mapping technique that makes static objects apparently move. Only by projecting a differential luminance motion pattern, the observer sees illusory movements in colorful pictures and real objects. This new technology is designed to cheat the observer’s brain based on the scientific knowledge about motion detection, and integration of motion information with color and form information.

## Plasticity of the Human Visual System

**Maria Concetta Morrone**

Department of Translational Research on New Technologies in Medicine and Surgery, University of Pisa – Fondazione Stella Maris IRCCS, Italy

The brain is flexible, and continuously adapts with experience, molding cortical circuitry to optimally analyze incoming information. These processes can be clearly demonstrated using multisensory stimuli, where the occipital cortical response to vision can be dramatically modulated or even annulled by competing sensory input from another modality. They can be also be demonstrated in patients with abnormal visual function such as amblyopes, the blind, and retinitis pigmentosa patients, or in patients who regained some vision with the ARGUS II Retinal Prosthesis. In all these cases, the adult brain has plastically adapted to the new aberrant visual information to optimize function. We have recently demonstrated that plastic changes in primary visual cortex are not limited to the developmental period, nor to pathological conditions, but can extend throughout life. Short-term monocular deprivation results in the deprived eye dominating rivalrous perception, lasting up to 3 h after patch removal, by reducing GABAergic inhibition in occipital cortex, demonstrated by MR spectroscopy at 7 T. Interestingly, the effect of short-term monocular deprivation is further enhanced by 15% by moderate levels of physical activity. As boosting brain plasticity is fundamental for the treatment of amblyopia, especially in adulthood, we recently attempted a counterintuitive experiment where we deprived the amblyopic instead of the dominant eye, to promote the recovery of visual function in adult amblyopic patients, combining monocular patching and physical activity. Strikingly, all our subjects showed an improvement of visual acuity, and 70% of them of stereopsis. Importantly, the improvement was preserved for at least 1 month after training. These results demonstrate that amblyopic vision can be improved by transiently depriving the weak rather than the strong eye, probably by activating homeostatic plasticity. Physical exercise may be crucial for the recovery by potentiating the plastic potential of the visual cortex as observed in animal models.

## The Evolution of Colour Vision in Ancient Predators

**Shaun P. Collin**

UWA Oceans Institute, School of Animal Biology, University of Western Australia, Australia

Meeting the challenge of visually sampling an ancient aquatic landscape by the early vertebrates was crucial to their survival and would establish a visual bauplan to be used by all subsequent vertebrate descendants. Image-forming eyes were under tremendous selection pressure, and the ability to identify suitable prey and detect potential predators was thought to be one of the major drivers of speciation in the Early Cambrian. Based on anatomical, electrophysiological, molecular and behavioural studies of extant representatives of the earliest stages in vertebrate evolution including jawless fishes (hagfishes and lampreys) and the first jawed fishes (sharks and their relatives), I will present predictions of the evolution of both colour and dim light vision in the context of visual predation. The complex and different chromatic sampling strategies of these ancient predators continues to provide important information regarding the selective pressures acting on different parts of the visual system and allows us to make more informed predictions of the visual ecology of ancestral vertebrates. I will also provide a couple of examples of how research on the visual system of some of these ancient predators (sharks) can be used to change natural behaviour with respect to the development of visual shark deterrents.

## Sessions

## Faces & Bodies I: Perception of Social Information

## The Development of Facial Expression Recognition

**Megan L. Willis^1^, Nicholas A. Badcock^2^, Nicole J. Ridley^3^ and Romina Palermo^4^**

^1^School of Psychology, ARC Centre of Excellence in Cognition and its Disorders, Australian Catholic University, Sydney, Australia; ^2^ARC Centre of Excellence in Cognition and its Disorders, Department of Cognitive Science, Macquarie University, Sydney, Australia; ^3^School of Psychology, Australian Catholic University, Sydney, Australia; ^4^School of Psychology, ARC Centre of Excellence in Cognition and its Disorders, University of Western Australia, Perth, Australia

Perceptual processing and emotional conceptual knowledge are key processes involved in the recognition of facial expressions, which develop over the course of childhood, along with facial expression recognition abilities. However, it is unclear whether these skills develop in a similar manner to facial expression recognition abilities. Twenty-six children (7–8 years), 20 adolescents (12–14 years), and 24 adults (18–35 years) completed a series of tasks assessing face identity matching, facial expression matching, emotional conceptual knowledge, and facial expression recognition. Children displayed poorer performance than both adolescents and adults on all tasks. Non-parametric correlational analyses revealed that greater performance on matching tasks (identity and expression) and emotional conceptual knowledge was associated with superior facial expression recognition performance. These relationships weakened in adolescence and adulthood. The results suggest that the development of facial expression recognition abilities in childhood is strongly linked to the development of perceptual skills and emotional conceptual knowledge.

## The Influence of Affective States on Working Memory Capacity of Biological Movements

**Fangfang Qiu, Rende Shui, Shulin Chen, Mowei Shen and Zaifeng Gao**

Department of Psychology and Behavioral Sciences, Zhejiang University, China

Biological movements (BMs) broadly refer to the movements of animate entities. Recent studies have begun to reveal the underlying mechanisms of holding BM information in working memory (WM); however, no study so far has investigated the interaction between affect and holding BM in WM. Here, we explored this issue by investigating the influence of happy, neutral, and negative affect in holding BM in WM. In Experiment 1, we required participants to remember 2–5 BM in a change-detection task after inducing different affective states. We found that WM capacity of BM was significantly dropped in the negative affect condition than in the happy or neutral affect condition, and no difference was found between the latter two. The reduction of BM capacity led by the negative affect was further confirmed in Experiment 2, by using an EEG index of mu-suppression. The current study suggests that negative affect reduces WM capacity of BM.

## Emotion Adaptation in the Peripheral Visual Areas Is Stronger Than in the Fovea

**Edwin Burns, Hui Chen Leong, Alice Chan and Hong Xu**

Nanyang Technological University, Singapore

Previous work has found that low-level adaptation in the visual periphery generates stronger aftereffects than in the fovea. However, this effect has yet to be confirmed in high-level emotion adaptation. Here, we examine emotion adaptation with respect to its adaptation eccentricity. Participants made emotion judgements to faces after adapting to a happy face or no adaptation (baseline); all of these faces were presented in one of nine locations across the horizontal visual field (VF). Faces appeared sadder in all locations after adapting to a happy face: the emotion adaptation aftereffect. The magnitudes of these aftereffects were only greater in the right VF, relative to centre. Without adaptation, faces presented in the left, but not the right, VF were perceived as sadder versus faces in the centre, thus driving the asymmetry in aftereffect magnitudes in the two VFs. These findings suggest that emotion adaptation is stronger in peripheral areas.

## Abnormal Emotion Adaptation With or Without Awareness in Developmental Prosopagnosia

**Edwin Burns^1^, Joel Martin^2^, Alice Chan^1^ and Hong Xu^1^**

^1^Nanyang Technological University, Singapore; Swansea University, UK; ^2^Nanyang Technological University, Singapore

Can a subcortical route process emotional faces’ holistic information without awareness or input from the fusiform face area (FFA)? Individuals with developmental prosopagnosia (DP) exhibit abnormalities in their FFA’s sensitivity to the holistic configuration of faces. Here, we adapted a group of DP cases and neurotypical controls (NT) to expressionless faces, happy faces and hybrid faces. Despite these hybrid faces being identified as expressionless due to their higher spatial frequencies taken from a neutral face, their low-spatial frequencies (LSF) conveyed holistic happy face information. Only NT produced emotion adaptation aftereffects without awareness to the hybrid faces; as a group, those with DP did not. By contrast, all participants exhibited emotion adaptation aftereffects to the intact happy faces, albeit this effect was smaller in DP. These results suggest that a neurotypically functioning FFA is required to process the LSF of emotion with or without awareness.

## Modelling Chinese and British Perceivers’ Facial First Impressions Using a Data-Driven Approach

**Clare Sutherland^1^, Xizi Liu^2^, Yingtung Chu^2^, Julian Oldmeadow^3^ and Andrew Young^2^**

^1^ARC Centre of Excellence in Cognition and its Disorders, School of Psychology, University of Western Australia, Australia; ^2^University of York, UK; ^3^Swinburne University of Technology, Australia; University of York, UK

People readily form impressions from facial appearance, and these judgements predict important social outcomes, such as election results, partner selection and financial lending. Studies with Western perceivers have found that three dimensions subserve their first impressions of Caucasian faces: approachability, youthful-attractiveness and dominance.

These three dimensions have been argued to relate to evolutionarily adaptive threat detection and mate choice, making it likely that these are universal dimensions of social face perception. However, models of facial impressions have yet to be built for non-Western perceivers. Here, we built data-driven models of facial impressions across perceiver culture and face race for the first time, by sampling spontaneous impressions made by Chinese and British perceivers. Approachability and youthful-attractiveness dimensions emerged from impressions across perceiver culture and face race, with some evidence of a third, capability dimension. Overall, the findings indicate that there is considerable cross-cultural agreement in dimensions underlying facial impressions.

## Testing the Dual-Route Model of Perceived Gaze Direction: Linear Combination of Eye and Head Cues

**Colin Clifford^1^, Yumiko Otsuka^2^ and Isabelle Mareschal^3^**

^1^The University of New South Wales, Australia; ^2^Ehime University, Japan; ^3^Queen Mary, University of London, UK

We have recently proposed a model which computes perceived gaze direction as a linear combination of eye and head direction. Here, we tested its adequacy by parametrically manipulating horizontal eye and head direction. Participants adjusted an on-screen pointer to indicate perceived gaze direction in two conditions: Normal and Wollaston. Images in the Normal condition included a change in the visible part of the eye along with the change in head direction, while images in the Wollaston condition were manipulated to have identical eye regions across head direction. Multiple regression analysis with explanatory variables of eye and head direction revealed that the linear model accounts for over 98% of the variance in each of the conditions. Further, introducing a non-linear term failed to explain significantly more variance. Thus, the current study supports the dual-route model that computes perceived gaze direction as a linear combination of eye and head direction.

## Temporal and Orientation Coding

## Individual Differences in Visual-Evoked Potentials Predict Temporal Sensitivity

**Brendan Keane^1^, Natasha Matthews^1^, Kielan Yarrow^2^ and Derek Arnold^1^**

^1^School of Psychology, University of Queensland, Australia; Department of Psychology, City University London, UK; ^2^School of Psychology, University of Queensland, Australia

The precision with which humans judge the relative timing of events is marked by considerable individual variance. Within a sample, it is not uncommon to find marked variation in the smallest audio-visual delay that can be discriminated, ranging from tens to hundreds of milliseconds. Understanding the basis of these individual differences may be of clinical significance, as impaired temporal sensitivity has been linked to conditions such as schizophrenia. We investigated these differences in a large sample of healthy adults using a combination of psychophysical tasks and EEG recordings of brain activity. We found that the precision with which observers judged the temporal order of audio and visual events was predicted by trial-by-trial variance in both the amplitude and latency of the visual N2 component (approximately 140 ms post stimulus-onset) and the visual P1 component (approximately 100 ms post stimulus-onset). Temporal sensitivity was not predicted by variance in the latency or amplitude of audio-evoked potentials. These data indicate that audio-visual timing sensitivity is limited by visual processing within cortex, particularly following initial stages of sensory activity.

## Temporal Processing Thresholds of Human Perception Correlate With Nonlinear VEP Responses in Cortex

**Alyse Brown^1^, Sheila Crewther^1^ and David Crewther^2^**

^1^La Trobe University, Australia; ^2^Swinburne University, Australia

Neuronal mechanisms behind perceptual processing speed are yet to be explored. Perceptual processing speed is based, in part, on the recovery speeds of the Magnocellular (M) and Parvocellular (P) visual pathways. In this study (*n* = 76), LED-driven achromatic flicker fusion thresholds for high (75%) and low (5%) contrasts were compared with the M and P nonlinearities of the multifocal visual-evoked potential (mfVEP), recorded from site Oz. Smaller nonlinearities are associated with greater neural efficiency in the visual system and the predominant M nonlinearity was predicted to negatively correlate with achromatic flicker fusion frequencies. As predicted, flicker fusion thresholds for high (*r* = −.319, *n* = 69, *p* < .003) and low (*r* = −.216, *n* = 68, *p* < .036) contrast negatively correlated with the amplitude of the primary M nonlinearity (K2.1 [N1-P1]) component, indicating that perceptual speed of processing is linked to more efficient cortical processing.

## Brightness Illusions in Toads and Humans Bring Focus Onto Retinal Function

**David Crewther^1^, Nina Riddell^2^, Laila Hugrass^1^, Ruxandra Martiu^1^, Eva-Louise Huber^1^, Aidan Holmes^1^, Jude Jayasuriya^1^ and Sheila G. Crewther^2^**

^1^Swinburne University of Technology, Australia; ^2^La Trobe University, Australia; Swinburne University of Technology, Australia; La Trobe University, Australia

Human psychophysics indicates that drifting gratings or diamonds shaded in a sawtooth pattern appear brighter when the direction of movement produces fast-OFF relative to fast-ON luminance profiles. Human pupillary function is similarly affected, with fast-ON stimulation resulting in pupil dilation and fast-OFF stimulation resulting in pupillary constriction. This suggests a retinal locus of the phenomenon. Retinal recordings of the trans-retinal DC potential support this idea. DC-ERGs revealed a sustained DC increase in trans-tissue potential during drifting patterns in general, plus a peak at drift offset. For sawtooth gratings, the DC potential effect is greater for fast-OFF cf. fast-ON sawtooth profiles. All gratings produced an increase in DC potential as temporal frequency increased. Pharmacological dissection of the DC potential effects was carried out with differences in response amplitude difference for the two sawtooth profiles remaining following ganglion cell (and spiking amacrine) suppression with Tetrodotoxin (TTX), blocking ON bipolars with 2-Amino-4-Phosphonobutyric acid (APB), and OFF bipolar cells with cis-Piperidine-2,3-dicarboxylic acid (PDA). Thus, human and toad sawtooth stimulus effects are consistent with a retinal source – in particular at the level of the photoreceptors.

## Preserved Feedback Connections in V1 Following Partial Vision Loss: Decoding Orientation From the Lesion Projection Zone in Individuals With Glaucoma

**Susan Wardle^1^, J. Brendan Ritchie^1^, Anina Rich^1^, Margery Pardey^1^, Stuart Graham^2^ and Mark Williams^1^**

^1^Department of Cognitive Science, Macquarie University, Australia; ^2^Faculty of Medicine and Health Sciences, Macquarie University, Australia; Department of Cognitive Science, Macquarie University, Australia

Attended stimulus features can be decoded from patterns of fMRI BOLD activation in regions of retinotopic visual cortex that do not receive direct feedforward inputs from the stimulus location. However, it is unknown whether attention-driven feedback signals to early visual areas require intact feedforward input. We attempted to decode the tilt of gratings from the scotoma lesion projection zone (LPZ) of V1 in five individuals with monocular glaucoma. Oriented gratings were presented in an intact visual field quadrant while subjects performed an orientation discrimination task. Significantly, for three-fifth patients, stimulus tilt could be decoded from both the stimulus quadrant and the LPZ quadrant, which received no feedforward input due to vision loss. These results suggest that attention-modulated feedback signals to early visual cortex do not require direct feedforward inputs, and further, that feedback connections to the LPZ may be preserved following partial vision loss in glaucoma.

## Tilted Lightness Illusions in an Exponential Filter Model

**Astrid Zeman^1^, Sennay Ghebreab^2^ and Kevin Brooks^1^**

^1^Macquarie University, Australia; ^2^University of Amsterdam, The Netherlands

Figures that induce lightness illusions, such as the Hermann Grid or the scintillating grid, are often rich in vertical and horizontal image structure. When these figures are tilted by 45°, a change in illusion magnitude is observed. Although spatial filtering models such as those based on Difference-of-Gaussians or Laplacian-of-Gaussian kernel functions can account for many lightness effects, they are isotropic in nature, producing orientation-invariant predictions. The exponential filter model, which has shown success in predicting lightness illusions, contains anisotropic filters that may be able to account for the orientation dependence of these lightness illusions. We use the exponential filter model to predict patch lightness for upright and tilted forms of lightness illusions and compare these predictions to human results.

## Symposia I: Sequential Effects in Face Perception

## Serial Dependency in Face Attractiveness Perception

**David Alais^1^, Gill Rhodes^2^ and Erik Van der Burg^3^**

^1^School of Psychology, University of Sydney, Australia; ^2^ARC Centre of Excellence in Cognition and its Disorders, School of Psychology, University of Western Australia, Australia; ^3^Department of Cognitive Psychology, Vrije Universiteit, The Netherlands

Sequences of brief stimuli can produce positive sequential dependencies where the preceding stimulus drives the current trial percept. This contrasts with repulsive aftereffects (negative dependencies) seen after sustained exposure to a single stimulus. We studied attractiveness perception using a large set of brief randomly interleaved male and female faces. We found strong positive dependencies: Current trial attractiveness judgements were higher for preceding attractive faces (and vice versa). This only occurred between consecutive same-sex faces, and scaled with face attractiveness increasing as the previous face’s attractiveness departed further from mean attractiveness. Effects were significant for 56 ms presentations, increased with duration to 250 ms, then plateaued for 500 and 1,000 ms. These results suggest a process which stabilises current perception by averaging the recent past. This would be beneficial for any attribute that remains largely stable over time (face attractiveness, identity, gender) but not for highly variable attributes such as expression.

## Different Coding Strategies for the Perception of Stable and Changeable Facial Attributes

**David Burr^1^, Jessica Taubert^2^ and David Alais^2^**

^1^Department of Neuroscience, University of Florence, Italy; ^2^School of Psychology, University of Sydney, Australia

Perceiving change can be fundamental; and adaptation mechanisms “which lead to negative aftereffects” optimize sensitivity to change. On the other hand, in a constant but noisy environment, integration of successive image views, which results in positive rather than negative serial dependencies, could be beneficial. What determines whether negative contrast or positive assimilation effects prevail? With morphing techniques we created stimuli varying simultaneously in gender (a stable attribute) and expression (a changeable attribute). Subjects scored each image in a random sequential presentation as male or female, and happy or sad. We found strong and consistent positive serial dependencies for gender, and strong and consistent negative serial dependency for expression. These results show that both positive and negative serial dependencies can operate at the same time, on the same stimuli, depending on the attribute being judged, pointing to very flexible and sophisticated optimization of past information.

## Race Categorisation: Sequential Dependency and Long-Term Adaptation

**Christopher P. Benton, Andrew L. Skinner and Annabelle S. Redfern**

School of Experimental Psychology, University of Bristol, UK

A person’s race affects their race categorisation judgements – a plausible example of real-world perceptual adaptation. We used a cognitive modelling approach to show that the standard repulsive effect of own race on race categorisation is driven by differential sensitivity to race-dependent perceptual cues. Asian participants were less sensitive to Asian cues than were our Caucasian participants (and vice versa). In contrast, analysis of sequential dependency showed a strong positive assimilation effect. Participants were more likely to respond “Asian” if the image to which they responded was preceded by a more Asian image. Preliminary analysis, using our cognitive modelling approach, indicates that this is caused by a difference in prior information as if participants simply assimilate the information from the previous image into their decision about the current image. These findings indicate that long term adaptation and sequential dependency show opposite effects that appear to be based on different mechanisms.

## Watching the Brain Recalibrate: An ERP Correlate of Renormalization During Face Adaptation

**Nadine Kloth^1^, Gill Rhodes^1^ and Stefan R. Schweinberger^2^**

^1^ARC Centre of Excellence in Cognition and its Disorders, School of Psychology, University of Western Australia, Australia; ^2^DFG Research Unit Person Perception, Department of Psychology, Friedrich Schiller University of Jena, Germany

Face distortion aftereffects have been proposed to result from renormalisation, which is difficult to observe during adaptation. Here, we establish the occipito-temporal P2 ERP as an indicator of renormalisation. Participants adapted to a sequence of four compressed or expanded adaptor faces and then classified a slightly compressed or expanded test face as undistorted or distorted. P2 amplitudes evoked by adaptors significantly increased over the sequence: They were smallest for the first adaptor and significantly larger for the second and third. Larger P2 amplitudes were also evoked by test faces for which adaptation had increased perceived normality. After adaptation to expansion, P2 amplitudes were larger for expanded than compressed test faces. After adaptation to compression, P2 amplitudes were larger for compressed than expanded test faces. We conclude that the P2’s sensitivity to the perceived deviation of a face from the norm makes the component an excellent tool to demonstrate adaptation-induced renormalisation.

## Motion Perception

## Binding Biological Motion and Location in Working Memory

**Zaifeng Gao, Quan Gu and Hong Ma**

Department of Psychology, Zhejiang University, China

Biological motion (BM) refers to the continuous configuration movement of human or animals in space. Binding BM to location in working memory (WM) has ecological significance since it allows us to know where the movement takes places and form coherent movement perception of others. However, so far, no study has explored the mechanisms of storing BM-location bindings in WM. Here, we addressed this issue by exploring (a) how many BM-Location bindings can be retained in WM; (b) whether involuntary object-based binding (e.g., retain BM while ignoring color) occurs for BM-location binding; c) how long does the object-based binding last in WM. In five behavioral experiments adopting change-detection task, we revealed that (a) Participants can retain 2–3 BM-location bindings; (2) object-based binding takes place automatically in WM; and (3) the object-based binding can be retained in WM for at most 3 s.

## Reduced Lag for Flashing Flash-Lag

**Hiroshi Ashida^1^ and Nicholas E. Scott-Samuel^2^**

^1^Graduate School of Letters, Kyoto University, Japan; ^2^School of Experimental Psychology, University of Bristol, UK

When a target is briefly flashed by a moving object, the former appears to be lagged behind even though the two are physically aligned. We found that this flash-lag illusion can be reduced when the moving object itself is flashing. We presented a white target above a horizontally moving red disk on a dark grey screen, and the luminance of the red one was sinusoidally modulated at 0, 4, or 8 Hz. The perceived spatial lag, measured by a method of constant stimuli, was reduced or even abolished when the moving disk was flashing. The result implies a potential risk of the flashing lights which are becoming popular among cyclists: While they might capture more attention from car drivers, and even though their perception could be more “veridical,” their reactions may be delayed when compared to a normal light.

## Global Motion Detection in Dogs (*Canis familiaris*)

**Orsolya Kanizsar^1^, Paolo Mongillo^1^, Gianluca Campana^2^, Luca Battaglini^3^, Anna Scandurra^1^ and Lieta Marinelli^1^**

^1^Laboratory of Applied Ethology, Department of Comparative Biomedicine and Food Science, Universita degli Studi di Padova, Italy; ^2^Department of General Psychology, Univerista degli Studi di Padova, Italy; ^3^Laboratory of Applied Ethology, Department of Comparative Biomedicine and Food Science, Universita degli Studi di Padova, Italy

The aim of our study was to determine thresholds of global motion perception in dogs and to investigate whether it could be enhanced by learning.

The procedure was based on simultaneous discrimination tasks of random dot displays presented on touch screens. Each dog (*N* = 3) underwent 400 trials (range of dot’s coherence 80%–20%) before (Experiment 1) and after (Experiment 2) being exposed 1,200 times to stimuli with variable dot’s coherence. Experiment 1 shows that threshold of global motion perception varied between 36.1% and 39.0% and after extensive exposure (Experiment 2) it remained unchanged in one dog, and dropped to 32.0% and 20.5% in the other two. Dogs’ perception threshold of global motion is similar or higher than that of other species (e.g., cats, humans), which questions the general claim on dogs’ higher performance in perceiving motion. Moreover, dogs’ perception of coherent motion can be enhanced by learning.

## Attenuated Global Biological Motion Adaptation in Autism Spectrum Disorders, and a Link to the Superior Temporal Sulcus

**Jeroen van Boxtel^1^, Steven Thurman^2^, Mirella Dapretto^2^ and Hongjing Lu^2^**

^1^Monash University, Australia; ^2^University of California, Los Angeles, USA

Biological motion research has shown that prolonged exposure to one action gives rise to an aftereffect that biases perception of a subsequently displayed action. Here, we investigated location-invariant (i.e., global) action adaptation, by adapting the visual system to biological motion at one location, and testing it at another location. Our sample included 18 typically developing children and 17 children with autism spectrum disorders (ASD). We found decreased location-invariant adaptation in children with ASD. We then investigated the neural origins of the location-invariant adaptation in a sample of typically developing adults. We found significant correlations between neural adaption in the superior temporal sulcus (STS) and behavioral adaption measures. The adaptation furthermore correlated with the level of autistic traits in this sample, consistent with our findings in ASD. We conclude that people with ASD show reduced global adaptation to biological motion, probably due to a reduced sensitivity in STS.

## Sensitivity and Bias in the Perception of Stream-Bounce Stimuli

**Mick Zeljko and Philip Grove**

University of Queensland, Australia

Stream-bounce refers to the resolution of ambiguous motion sequences as “streaming” or “bouncing” depending on the presence or absence of a sound. We used signal detection theory to determine its sensory or decisional origins. We measured observers’ sensitivity and criterion when detecting a weak auditory signal, concurrent with objectively streaming or bouncing displays. Observers’ criterion was more liberal with bouncing targets than for streaming targets with no change in sensitivity. We next tasked participants to detect a weak tone in noise while viewing ambiguous motion sequences. They also indicated whether the targets appeared to stream or bounce.

Observers’ sensitivity and criterion measures for detecting sounds were inconsistent with sensory factors determining the resolution of stream-bounce displays. Moreover, observers reported bouncing on both hit and false alarm trials and streaming for both correct rejection and miss trials. These results support later decisional factors over early sensory factors underlying the effect.

## Stereoscopic Advantages for Vection Induced by Radial, Circular and Spiral Optic Flows

**Stephen Palmisano^1^, Stephanie Summersby^1^, Rodney Davies^1^ and Juno Kim^2^**

^1^The University of Wollongong, Australia; ^2^The University of New South Wales, Australia

Although observer motions present different patterns of optic flow to our left and right eyes, researchers have mostly ignored any potential stereoscopic contributions to self-motion perception. Here, we investigated the effects on visually induced illusory self-motion (i.e., vection) of adding consistent stereoscopic cues to radial, circular and spiral optic flows. Under natural viewing conditions, we found strong stereoscopic vection advantages for both radial and spiral, but not circular, flows. However, a stereo vection advantage was observed for circular flows when monocular motion signals were weakened by limiting dot lifetimes. Our primary finding was that the stereoscopic vection advantage for spiral flow was significantly larger than that obtained for radial flow. This finding may be explained by comparable differences in motion aftereffect duration for these displays, which suggests that global circular motion selectively reduces adaptation to stereomotion in the case of spiral optic flow.

## Local and Global Form

## Spatial Frequency Contributions to Global and Local Processing in Hierarchical Figures

**Branka Spehar, Luke Vu and Tam Mai**

School of Psychology, UNSW, Australia

Most, if not all, visual input is hierarchically organised, consisting of both local and global levels of information. Global precedence refers to the dominance of the global over local level of processing under many conditions. Although the contribution of low-spatial frequency mechanisms in the prioritisation of global level processing has long been recognised, the spatiotemporal characteristics of this contribution remain unclear. We investigate the interaction between spatial frequency mechanisms and hierarchical processing in typical individuals and in individuals with high level of self-reported autistic-like traits (AQ). Consistent with the previous findings, high AQ observers exhibited local interference with full spectrum displays. However, high AQ observers displaced reduction in global interference, particularly in the low-spatial frequency displays. The reduced spatial integration in the low-spatial frequency channels is likely to contribute to many findings of atypical visual performance that requires attention to a local signal while ignoring global noise.

## Textured Paths: Contours or Textures?

**Ken Tan, Edwin Dickinson and David Badcock**

School of Psychology, University of Western Australia, Australia

Radial Frequency (RF) patterns and RF textures have been previously shown to be globally processed. RF textures required the impression of a closed flow (flowsure) for integration while RF patterns did not. In this study, we investigate whether the application of an RF texture to an RF pattern would cause the resultant contour to be processed as a texture or contour. Flowsure was not required for the textured RF paths to be integrated, indicating they were processed as second-order paths. Second-order paths were observed to have discrimination thresholds approximately double those for first-order paths, and we postulated if this was due to double the amount of information in the first-order stimulus. Whether the benefit was due to an increased cue benefit and how those cues combined was then investigated. We observed that when utilized on the same contour, first- and second-order cues combine linearly for global pooling of shape.

## The Effect of Contrast Gain Control on Perceptual Grouping

**Chien-Chung Chen and Yih-Shiuan Lin**

Department of Psychology, National Taiwan University, Taiwan

To estimate the contrast gain control mechanisms underlying perceptual grouping, we used a variant of glass patterns that composed of randomly distributed tripoles. Each tripole contained an anchor dot and two context dots. Linking the anchor to one of the context dot would produce a global percept of a clockwise (CW) spiral while linking to the other dot, a counterclockwise (CCW) spiral. We manipulated the chromaticity and contrast of each dot. The observer was to determine whether the spiral was CW or CCW. In the isochromatic conditions, the probability of grouping one context dot with the anchor was an inverted-U shape function of the context dot contrast with the peak position increased with the contrast of the other context dot. In the isoluminance condition, such probability increased monotonically with the context dot contrast. Our result cannot be explained by existing models for perceptual grouping but a divisive inhibition model.

## Orientation-Specific Excitatory and Inhibitory Gain Influences on a Bank of Orientation Selective Channels Predict the Effects of Orientation Context

**Edwin Dickinson, Olivia Tan and David Badcock**

School of Psychology, The University of Western Australia, Australia

Effects of orientation in spatial context can be repulsive in the sense that the orientation of a test line is misperceived as exaggerated in its difference from the orientation of a contemporaneously presented context line. The orientation of a test line composed of oriented elements is, however, attracted to the orientation of those elements, which might also be considered an orientation context. In each case, the orientations of test and context are available to perception simultaneously and so the analysis of each must be discrete. The context effects can be accounted for by orientation specific inhibitory and excitatory influences of the context on a bank of orientation selective information channels encoding the orientation of the test. We show that the context effects can be induced together and that the perceived test orientation is predicted by the vector sum of the response of the bank of channels in a double-angle space.

## Looking for Symmetry: Fixational Eye Movements Are Biased by Image Mirror Symmetry

**Jason Bell^1^, Andrew Isaac Meso^2^, Anna Montagnini^3^ and Guillaume S. Masson^3^**

^1^School of Psychology, University of Western Australia, Australia; ^2^Psychology & Interdisciplinary Neuroscience Research Group, School of Science and Technology, Bournemouth University, UK; ^3^Institut de Neurosciences de la Timone, CNRS & Aix-Marseille Universite, France

Human vision is highly sensitive to mirror symmetry. We characterised patterns of fixational eye movements made by observers staring at synthetic scenes either freely (i.e. free exploration) or during a symmetry orientation discrimination task (i.e. active exploration). Stimuli could be mirror-symmetric or not. Both free and active exploration generated more saccades parallel to the axis of symmetry than along other orientations. Most saccades were small (<2°) leaving the fovea within a 4° radius of fixation. The analysis of saccade dynamics showed that the observed parallel orientation selectivity emerged within 500 ms of stimulus onset and persisted throughout the 3-s trial, under both viewing conditions. Symmetry strongly distorted existing anisotropies in gaze direction in a seemingly automatic process. We argue that this bias serves a functional role in which adjusted scene sampling enhances and maintains sustained sensitivity to local spatial correlations arising from symmetry.

## Shading Cues for Perceiving Translucency

**Juno Kim^1^, Phillip Marlow^2^ and Barton Anderson^2^**

^1^School of Optometry and Vision Science, The University of New South Wales, Australia; ^2^School of Psychology, University of Sydney, Australia

Visual perception of surface and materials (e.g., shape, reflectance and opacity) requires the classification of luminance variations of images into multiple physical causes. Previous studies have shown that the proximity of specular reflections relative to brighter regions of diffuse shading is important for the perception of gloss. Shading for glossy opaque surfaces normally decreases as a function of surface orientation away from the primary lighting direction. Here, we show that partial violation of this constraint can alter the appearance of a surface’s translucency. We find that perceived translucency can be generated by increasing the shading intensity of surface regions contralateral to the specular highlights.

This effect of shading on perceived translucency was not explained by a general decline in shading contrast, even when we considered images matched in perceived shading contrast. The results suggest that perceived translucency depends on the structure of shading flow relative to position of specular highlights.

## Developmental Disorders and Aging

## Face Recognition Accuracy, Response Time and Visual Scanning Behaviour of Adolescents With and Without Autism Spectrum Disorders

**Julia Tang, Marita Falkmer and Torbjorn Falkmer**

School of Occupational Therapy and Social Work, Curtin University, WA, Australia

Few studies have evaluated the impact of the possible variation in face recognition ability along the developmental trajectory in autism spectrum disorders (ASD). The current study recruited 28 adolescents with ASD and 30 matched typically developing (TD) peers.

Participants viewed 12 pairs of face stimuli cut into puzzle pieces (encoding), followed by a face recognition phase. Measurements of visual scanning behaviour, that is, number of fixations and fixation duration were recorded using an eye tracker. Adolescents with ASD demonstrated increased difficulty in face recognition compared to their TD counterparts, despite showing similar response time. Adolescents with ASD were less likely to derive effective processing using the “face information triangle,” as reduced scanning to the eyes but increased on other areas of the face were observed. Overall, this study highlighted that face recognition differences between individuals with and without ASD first appear during adolescence.

## Accuracy, Response Time and Visual Search Strategies of Adolescents With and Without Autism Spectrum Disorder During a Disembedding Task

**Melissa Black, Marita Falkmer, Sonya Girdler and Torbjorn Falkmer**

School of Occupational Therapy and Social Work, Curtin University, WA, Australia

Children with ASD process information differently in visuo-spatial tasks. The current study employed a standardized assessment of visual perceptual skills of children with ASD to investigate disembedding performance of 27 adolescents with ASD and their matched peers. The accuracy, response time and visual search strategies were recorded by an eye tracker. The aim of the study was to examine performance and visual search strategies of adolescents with and without ASD to provide insights in to the theories of Weak Central Coherence (WCC) and Enhanced Perceptual Functioning (EPF). Adolescents with ASD were found to be slower at completing the disembedding task with the sole differences in visual search between groups relating to the number of fixations and the duration of first fixations outside of the figures, that is, redundant space. These results provide limited evidence for a local preference in ASD and contradict the WCC and EPF theoretical viewpoints.

## Concept, Creativity and Complexity in Autism Spectrum Disorders: A Study Using the Vygotsky Blocks

**Paul Constable^1^, Melanie Ring^2^, Sebastian Gaigg^2^ and Dermot Bowler^2^**

^1^Flinders University, Australia; ^2^City University London, UK

This study explored the Vygotsky Block Test (VBT) in assessing how individuals with autism spectrum disorder (ASD) used convergent and divergent thinking strategies to define new concepts when building categories from a set of novel, multidimensional stimuli.

The ASD group: *N* = 23 (21 male, 2 female, *M* age = 40.4 years, range: 26–66 years) and the TD group: *N* = 24 (22 male, 2 female, *M* age = 38.7 years, range: 22–65 years). Groups were matched on gender and Wechsler Adult Intelligence Scales.

There were significant group differences between the ASD and TD participants on their ability to use a convergent thinking strategy (*p* < .05) to correctly categorize the blocks as well as in divergent thinking (*p* < .01) when defining new groupings from the blocks.

The findings support use of the VBT as a method for exploring concept formation during categorization and the use of inner-dialogue in ASD.

## Age-Related Differences in Perceptual Motion Suppression Correlates With Gamma Aminobutyric Acid (GABA) Concentration in Visual Cortex

**Allison M. McKendrick^1^, Kabilan Pitchaimuthu^1^, Qizhu Wu^2^, Olivia Carter^3^, Bao N. Nguyen^1^ and Gary Egan^2^**

^1^Department of Optometry & Vision Sciences, The University of Melbourne, Australia; ^2^Monash Biomedical Imaging, Monash University, Australia; ^3^Melbourne School of Psychological Sciences, The University of Melbourne, Australia

Normal aging reduces perceptual motion suppression which has been attributed to presumed age-dependent reductions in levels of the inhibitory neurotransmitter GABA. We tested this hypothesis by measuring GABA in visual cortex using magnetic resonance spectroscopy and perceptual motion suppression in the same people. GABA concentration was estimated using MEGA PRESS in a Siemens Skyra 3T scanner with 32-channel head coil. GABA/tCr was adjusted for grey matter volume. Twenty older (63–78 years) and twenty younger (20–37 years) adults participated. Motion suppression was estimated by measuring duration thresholds as described by Tadin et al. (Nature, 2003). In line with previous findings, older adults showed reduced motion suppression, *t*(38) = 5.438, *p* < .01. Unexpectedly, however, they had elevated GABA in visual cortex, t(38) = −4.209, *p* < .01, with the two variables negatively correlated (r = −.483, *p* < .01). Our data are inconsistent with a simplistic model of reduced GABAergic inhibition in single neurons governing perceptual motion suppression.

## Normal Aging Affects Visual Contextual Effects of Orientation, Contrast, Flicker and Luminance

**Bao N. Nguyen and Allison M. McKendrick**

Department of Optometry and Vision Sciences, University of Melbourne, Australia

Previous studies show that aging alters certain visual spatial contextual effects with particular emphasis on tasks involving manipulations of contrast and motion. Here, we aimed to determine whether aging alters other contextual effects (e.g., involving luminance, flicker, orientation), and whether the strength of effect is similar across all tasks. Previous literature suggests that pre-cortical neural circuitry is sufficient to enable encoding of centre-surround luminance and flicker effects, whereas properties of contrast and orientation contextual effects are more consistent with additional cortical computation. We compared the perceived luminance, flicker, contrast, and orientation of a central target with and without a surround in 18 younger (19–31 years) and 18 older adults (60–75 years). Older adults showed stronger contextual effects than younger observers (main effect of group *p* < .001) of a similar magnitude across all tasks (interaction between group and task *p* = .26), indicating that normal aging results in widespread alterations to spatial contextual processing.

## Attention, Clinical Disorders, and Form

## To the Right or to the Left: Left Superiority for Multiple-Feature Attentional Selection

**Shih-Yu Lo**

National Chiao Tung University, Taiwan

In this study, I examine the efficiency of attentional selection on two stimuli that share the same or different features. The observers were presented multiple groups of dots in the display. The task was to select one group of dots on the left side and the other group on the right side, and to detect a speed change with varying duration. The two selected groups of dots could move in the same or opposite directions. The results suggest that when the selected groups of dots moved in the same direction, there was little difference between their selection efficiencies between the stimuli on the two sides. However, when the selected groups of dots moved in opposite directions, the selection efficiency for the left group of dots was higher than for the right group. These results suggest that the right hemisphere has an advantage for selecting stimuli within the left visual field than the left hemisphere as to the right visual field. However, this asymmetry in selection efficiency could be evened out by feature similarity of the two selected groups.

## Pleasant and Unpleasant Sounds and Visual Processing of Neutral Targets in Lower and Upper Visual Space

**John McDowall and Rebekah Smith**

Victoria University of Wellington, New Zealand

In this study, we examined whether pleasant non-word sounds (e.g. laughter) or unpleasant non-word sounds (e.g. screaming), would influence visual spatial attention in a vertical manner. Based on previous work using valenced words, pictures and faces, we hypothesized that pleasant sounds would direct attention upwards in visual space, and unpleasant sounds would direct attention downwards in visual space. Eighty-three participants listened to sound bites either pleasant or unpleasant and then identified targets either at the top or bottom of a computer screen. The results of this study did not show a bias in spatial attention. Possible reasons are discussed.

## Division of Spatial Attention Examined by Evoked Potentials

**Takumi Miura^1^, Kazumichi Matsumiya^2^, Ichiro Kuriki^2^ and Satoshi Shioiri^2^**

^1^Graduate School of Information Science, Tohoku University, Japan; ^2^Research Institute of Electrical Communication, Tohoku University, Japan

To investigate whether visual attention can be divided into multiple spatial locations or not has been debated for decades, but it is still an open question, we estimated spatial modulation of visual attention by using SSVEP (Steady-State Visual Evoked Potential) and ERP (Event Related Potential). EEG was measured while subjects paying attention to detect targets presented simultaneously at two of eight locations. The spatial profile of P3 in ERP response showed inhibition between the two attended locations although SSVEP did not. Since there is no difference in the inter-event variance of P3 between one target and two target presentations, it is unlikely that attention shifted in time between the two attended locations. These results, together with SSVEP results, may suggest that there are different attentional mechanisms, one with ability for division and the other with unitary spotlight of visual attention.

## Threatening Faces Fail to Guide Attention for Adults With Autistic-Like Traits

**Michael C. W. English, Troy A. W. Visser and Murray T. Maybery**

School of Psychology, The University of Western Australia, Australia

Individuals diagnosed with autistic spectrum conditions often show deficits in processing emotional faces relative to neurotypical peers. However, little is known about whether similar deficits exist in neurotypical individuals who show high levels of autistic-like traits. To address this question, we compared performance on an attentional blink task in a large sample of adults who showed low or high levels of autistic-like traits on the Autism Spectrum Quotient. We found that threatening faces inserted as the second target in a rapid serial visual presentation were identified more accurately amongst individuals with low compared to high levels of autistic-like traits. This is the first study to show that attentional blink abnormalities seen in autism extend to the neurotypical population with autistic-like traits, suggesting that at least with respect to attentional mechanisms, there is considerable overlap between individuals diagnosed with autism and neurotypical individuals with high levels of autistic-like traits.

## Misperceiving Size: Serial Dependence in Female Body Perception

**Joanna Alexi^1^, Romina Palermo^1^, Nadine Kloth^1^, Sue Byrne^2^, Dominique Cleary^2^, Kendra Dommisse^2^ and Jason Bell^1^**

^1^ARC Centre of Excellence in Cognition and its Disorders, School of Psychology, University of Western Australia, Australia; ^2^School of Psychology, University of Western Australia, Australia

Attentional biases to certain body types, or to specific body parts, are associated with high levels of eating disorder symptomology, indicating the importance of body perception. The current research examined the veridicality of body perception, using both real body images and synthetic, computer-generated body images. The latter provides a test of the validity of using synthetics to represent extreme body shapes in particular. Participants briefly (250 ms) viewed a single body image that could range from severely underweight to obese and were asked to judge the body type using a visual analogue scale. Results showed that, for both real and synthetic bodies, the perception of body size is biased towards previously viewed body shapes, the first empirical evidence of positive serial dependence in body perception. The relationship between body size misperceptions and eating disorder symptomology is discussed, to further our understanding of the functional role of serial dependencies in vision.

## The N2pc Component and Inhibition of Return

**Alfred Lim, Vivian Eng, Shamsul Azrin Jamaluddin and Jason Satel**

University of Nottingham Malaysia Campus, Malaysia

Inhibition of return (IOR) is characterized by delayed responses to previously attended locations when the time interval between stimulus onset is long enough. In a typical cue-target paradigm, a manual or saccadic response is required when a target stimulus is presented at the same (cued) or opposite (uncued) location as the cue. In this series of experiments, we paired the target with a distractor at the opposite location, providing balanced sensory stimulation at the time of target onset. This design allowed analysis of the N2pc component, an event-related potential associated with target detection in visual search paradigms. This component has been largely overlooked in EEG studies of IOR due to the use of unbalanced designs. Results showed that an N2pc component was present for both cued and uncued trials, with a large cueing effect (smaller N2pc amplitude for cued trials), likely associated with the exhibited behavioural IOR effect.

## Separating Decision Criteria Between Two Independent Visual Searches Is Difficult If They Are Perceptually Similar

**Han-Gyeol Son, Hyung-Bum Park and Joo-Seok Hyun**

Department of Psychology, Chung-Ang University, South Korea

The probability of target presence in visual search (i.e., target prevalence) has been known to influence the likelihood of target detection and the speed of search termination. Here, we report that biasing decision criteria in a search by manipulating its target prevalence influences performance for another search without such prevalence. Participants performed either of two independent search tasks across trials where one had the prevalence of 10, 50, or 90% while the other had 50% (i.e., neutral). The prevalence-search influenced performance of the neutral-search when the items between them were identical but did not influence when different. Further tests revealed that the perceptual similarity of the items primarily determined the transferability of target prevalence effect between the searches rather than the shared target feature. These results indicate that observers often fail to separate their decision criteria between two independent but concurrent visual searches if their search items are perceptually similar.

## Non-Conscious Local and Global Cueing Differences in Subthreshold ASD Traits: A Continuous Flash Suppression Study

**Robin Laycock, Daniel Chan and Sheila G. Crewther**

School of Psychology and Public Health, La Trobe University, Australia

Previous research suggests that local/global visual perceptual abnormalities characterize autism spectrum disorders (ASD). In ASD, there is also evidence for impairments in sub-cortical networks subserving emotion processing, although these regions are now also recognized as being involved in (non-emotional) salience detection. We examined non-conscious processing of local/global arrow stimuli in neurotypical participants with varying ASD-traits by utilizing Continuous Flash Suppression of a Posner cuing paradigm. The cueing effect (reaction time for valid subtracted from invalidly cued trials) was compared between high and low ASD-trait groups. While low ASD-trait participants demonstrated a globally biased cueing effect, high ASD-trait participants demonstrated a locally biased cueing effect, indicating that local/global anomalies in ASD might also be processed without conscious awareness.

## High Schizotypy Shows Superior Putative Subcortical Magnocellular-Driven Emotion Processing and Deficits in Cortical Attentional Change Detection

**Robin Laycock, Liz Cutajar and Sheila G. Crewther**

School of Psychology and Public Health, La Trobe University, Australia

Visual processing deficits have been established in schizophrenia patients, and also in relatives and individuals with high schizotypy traits. Neurotypical participants with varying self-reported schizotypy personality traits (SzP) were compared on visual tasks, including a face emotion discrimination task with conditions targeting low (magnocellular) or high (parvocellular) spatial frequency channels. High SzP participants showed superior magnocellular processing (flicker-defined form contrast sensitivity), but inferior dorsal stream and transient attention processing (motion coherence, change detection) compared with low SzP participants. Both groups showed reduced accuracy for disgust compared with neutral/happy emotion discrimination; however, high SzP participants showed greater accuracy for low spatial frequency disgust emotions compared with low SzP participants.

Schizotypy may thus be associated with superior magnocellular processing, but deficits in dorsal stream/transient attention, possibility implicating routes bypassing V1 in schizotypy.

## Visual Acuity and Contour Interaction With Luminance-Modulated and Contrast-Modulated Noise Stimuli in Adults With Monocular Defocus

**Pui Juan Woi, Mohd Izzuddin Hairol and Sharanjeet Kaur**

Optometry & Vision Sciences Programme, Universiti Kebangsaan Malaysia, Kuala Lumpur, Malaysia

Contrast-modulated (CM) stimuli are thought to be processed in more binocular areas than those processing luminance-modulated (LM) stimuli, and may be prone to deficits in amblyopic visual systems. Some visual deficits in amblyopic eyes can be modelled by imposing blur in healthy individuals. We compared acuities and contour interaction effects using LM and CM letters, measured binocularly in five normal adults under monocular defocus (0-4 D). Across all levels of monocular defocus, (a) binocular acuity for LM letters was ∼2.9’—better than that for CM letters (*p* < .05), (b) contour interaction magnitude was similar for both stimulus types (*p* > .05), and (c) contour interaction extent in arcmin was ∼2’—larger for CM stimuli than that for LM stimuli (*p* < .05). Monocular defocus degrades binocular acuity and performance of LM and CM stimuli similarly, which suggests that monocularly blurring a visual system with normal binocular vision may not fully reveal visual degradations in amblyopia.

## The Contribution of Attention and Anxiety to the Neurodevelopmental Phenotype

**Alana Cross, Robin Laycock, Jessica Peters and Sheila Crewther**

La Trobe University, Melbourne, Australia

It is widely acknowledged that visual differences exist across the autism spectrum. However, the literature has not yet adequately considered the interactive contributions of anxiety and attention to behavioural problems noted in children with neurodevelopmental disorders.

Forty primary school children aged between 5 and 12 years were compared between groups with high and low parent-rated autistic tendency and anxiety using the Autism Spectrum Quotient-Children’s Version (AQ-Child) and Spence Children’s Anxiety Scale (SCAS). Children with high autistic tendency displayed greater sensitivity on a flicker contrast task, highlighting magnocelluar system involvement in perceptual differences. No group differences were established on an inspection time task. The effect of anxiety as a mediating factor between ASD-traits and visual processing was examined. A neurodevelopmental phenotype appears to be characterised by heightened anxiety and attentional differences that likely contribute to social and learning difficulties.

## Searching for Closed-Contours and Schizotypy

**Kirsten R. Panton^1^, Johanna C. Badcock^2^, J. Edwin Dickinson^1^ and David R. Badcock^1^**

^1^School of Psychology, University of Western Australia, Australia; ^2^Centre for Clinical Research in Neuropsychiatry, School of Psychiatry and Clinical Neurosciences, University of Western Australia, Australia; Cooperative Research Centre – Mental Health, VIC, Australia; School of Psychology, University of Western Australia, Australia

Impairments in perceptual organization have been reported in people with schizophrenia and in healthy schizotypes, considered at increased risk for schizophrenia. These impairments have traditionally been examined with the Embedded Figures Test (EFT), and interpreted as an alteration in global rather than local processes. Criticisms of the EFT led to the development of the Radial Frequency Search Task (RFST), which can more reliably distinguish between these processes, but has not previously been applied to schizotypy groups. Students with high (*n* = 54) and low (*n* = 124) levels of schizotypal traits completed the EFT and two RFSTs which selectively targeted global and local processes.

Unexpectedly, there was no significant difference between groups on the EFT. In contrast, on the RFST the high schizotypy group was slower and less efficient on the global but not the local processing task. These findings suggest a specific difficulty in global processing of visual stimuli in high schizotypes.

## Interocular Suppression Reduces Perceived Speed in Patients With Amblyopia and Normal Controls

**Goro Maehara^1^, Syunsuke Araki^2^, Benjamin Thompson^3^ and Atsushi Miki^2^**

^1^Kanagawa University, Japan; ^2^Kawasaki Medical School, Japan; ^3^University of Waterloo, Canada

We investigated the effect of interocular suppression on the relative perceived speed of dichoptically presented motion stimuli. The stimuli consisted of quadrants containing moving dots. One eye viewed the top-left and bottom-right quadrants, and the other eye viewed the top-right and bottom-left quadrants. Ten observers with normal vision and three amblyopes (one strabismic, one anisometropic, and one combined strabismic-anisometropic) matched the perceived speed of the stimuli presented to each eye. Normal observers viewed with a 2.0 ND filter in front of one eye to induce partial suppression.

Speed was increased in the filtered eye or decreased in the non-filtered eye to achieve equal speed perception between the eyes. The three observers with amblyopia also showed this interocular mismatch without an ND filter. These data suggest that partial interocular suppression decreases the perceived speed of suprathreshold stimuli seen by the suppressed eye.

## Is Global Integration of Contour Information Incremental or All-or-None?

**David R. Badcock, Kirsten R. Panton and J. Edwin Dickinson**

School of Psychology, The University of Western Australia, Australia

Radial frequency (RF) patterns are produced by sinusoidal modulation of the radius of the contour. Schmidtmann, Kennedy, Orbach, and Loffler (2012) proposed that the patterns were processed through “AND” gates, requiring all constituent cycles of an RF pattern to be visible before integration was detectable. Others have proposed that global integration is an incremental process with thresholds being proportional to the amount of contour modulated. These different outcomes may be associated with the smoothing contour used to return a modulated cycle to the original circular radius. The current study re-examined this idea using four expert observers. Psychophysical deformation discrimination thresholds improved as a power function of the number of modulated cycles. There was no sudden change when the pattern was completed. Local cues from an inappropriate smoothing function may explain the lower rate of improvement until the full stimulus is modulated in the Schmidtmann et al. study.

## Shape Distortion Illusion of a Dashed Line Circle Induced by Gradation Flash

**Kenzo Sakurai**

Department of Psychology, Tohoku Gakuin University, Japan

Visual illusions of shape distortion that circles turn into polygons (e.g., hexagons) have been reported with using a prolonged viewing in peripheral vision (Khuu, McGraw, & Badcock, 2002) and with using afterimages (Ito, 2012). Recently we found that the shape distortion illusion can be induced in a short period by presenting alternation of a circle and its inward gradation pattern (Sakurai, 2014 VSS, ECVP; Sakurai & Beaudot, 2015 VSS). In order to extend this study, we investigated whether a dashed line circle distorted with the gradation flash when the length of line segments was varied. Results of induction time (latency) and verbal reports showed that the shape distortion of a dashed line circle was induced, and the corners of polygon were aligned to the gaps of the dashed line. This suggests that curvature detectors involve the shape distortion effect.

Supported by JSPS Grant-in-Aid for Scientific Research (B) Grant Number 25285202.

## Evidence for Global Processing in Radial Frequency Patterns

**Robert Green, Edwin Dickinson and David Badcock**

School of Psychology, The University of Western Australia, Australia

Global processing (or additive summation) of a shape is the integration of local information around the shape’s contour aiding in its detection and discrimination. Recent research using Signal Detection Theory (SDT) has suggested that stimuli previously analysed using High Threshold Theory (HTT) and thought to be globally processed may in fact be a product of probability summation (increased likelihood of detection due to increased number of signal elements). Four expert observers (two naive) participated in detection and discrimination tasks involving radial frequency (RF) patterns. We compared detection and discrimination thresholds for both an RF3 and an RF5 and found evidence for global processing of both stimuli. The simple application of SDT did not support the conclusions reached from the data, suggesting it is an inappropriate method for analysing global integration in RF patterns. Both the method presented here and HTT did support the conclusion that shape information is globally integrated.

## Local and Global Contributions to Detection of Radial Frequency Patterns Across Development

**Serena J. Cribb^1^, Johanna C. Badcock^2^, Murray T. Maybery^1^ and David R. Badcock^1^**

^1^University of Western Australia, Australia; ^2^Centre for Clinical Research in Neuropsychiatry, School of Psychiatry and Clinical Neurosciences, University of Western Australia, Australia and Cooperative Research Centre – Mental Health, VIC, Australia

Previous studies have demonstrated that children are less sensitive to fully modulated RF patterns than adults, but this difference could arise from either changes in the extent of contour integration or sensitivity to local curvature information, or both. In the present study, psychophysical methods were used to separate changes in sensitivity to local curvature information from changes in contour integration. Typically developing observers were tested (aged 6–22, *N* = 104, normal acuity) on an RF integration task. Compared to older observers, younger observers were less sensitive to RF3 patterns in all conditions.

However, their strength of integration did not change with age. Results suggest changes in sensitivity to RF patterns across age are due to increased sensitivity to local curvature information rather than increased effectiveness of contour integration. We find this to be the case regardless of the method used for calculating probability summation.

## Attention: Memory and Bias

## Distractibility Across Vertical and Horizontal Space: Individual Differences in Visuospatial Attention

**Nicole A. Thomas, Ellie Aniulis and Michael E. R. Nicholls**

Flinders University, Australia

Healthy individuals show a reliable bias to the left, known as pseudoneglect. The magnitude of this attentional asymmetry varies depending on visual field location. We investigated whether brief visual distractors, which recruit exogenous attention, influence the strength of pseudoneglect. A vertical landmark task with horizontally presented distractors was also performed. Experiment 1 findings illustrated single visual field distractors led to stronger leftward biases, when compared to dual visual field distractors. A baseline landmark task was included in Experiment 2 to allow participants to be separated into left- and right-responders. Upper space distractors increased the magnitude of asymmetries depending on baseline direction; left-responders showed increased leftward biases and right-responders showed increased rightward biases. Upward biases on vertical lines were unchanged by horizontal distractors, and horizontal and vertical asymmetries were not correlated. These results confirm individual differences in pseudoneglect influence distractibility and also highlight the importance of accounting for baseline attentional asymmetries.

## Visual Memory Is the Expert’s Accompaniment to “Practice Makes Perfect”

**Kirsten Challinor, Sophia Tai and Patricia Arthur**

School of Optometry & Vision Science, UNSW, Australia

It takes more than 10,000 h of deliberate practice to become an expert (Ericsson et al., 1993); but practice alone does not an expert make (Hambrick et al., 2014). The mean VSTM score for 9 expert sight reading pianists (7.3 items, *SE* = 0.74) was higher than 12 non-expert sight readers (6.4 items, *SE* = 0.74), and this difference was significant, *t*(18) = 2.64, *p* < .05.

Furthermore, it was found that reading a simple, randomized word list aloud at speed was significantly faster in 8 experts (3.9 Words per second, *SE* = 0.39) than in 11 non-experts (3.1 Words per second, *SE* = 0.45): significant *t*(29) = 3.89, *p* < .001. Experiments using Chess players ranked for expertise were undertaken to test the validity of speed word reading and VSTM scores as a domain independent measure of visual processing expertise.

## Creating Objects in Visual Working Memory: Evidence From Object-Based Attention

**Haihang Zhang, Jifan Zhou, Rende Shui and Mowei Shen**

Department of Psychology and Behavioral Sciences, Zhejiang University, China

This study reports on how visual working memory (VWM) forms intact perceptual representations of visual objects using sub-object elements. We used both facilitation and interference effects of object-based attention to test whether sequentially encoded object fragments could be integrated into objects in VWM and found that when subjects’ attention was cued to a location occupied by the VWM object, the target presented at the location of that object was perceived as occurring earlier than that presented at the location of a different object (Experiment 1); responses to a target were significantly slower when a distractor was presented at the same location as the cued object (Experiment 2). These results suggest that object fragments can be integrated into objects in VWM with the law of visual perception. Thus, our finding provides new evidence for VWM interactive model from object level.

## Retaining Information in the Focus of Attention of Working Memory Requires Object-Based Attention

**Huayu Liao, Mowei Shen and Zaifeng Gao**

Department of Psychology and Behavioral Sciences, Zhejiang University, Hangzhou, China

By using a retro-cue task of working memory (WM), researchers have consistently found that information in the focus of attention (FoA) is in a highly activated state in WM, and we first extract the information in the FoA in the ongoing cognitive processing. Although extensive studies have been conducted in exploring the mechanisms of FoA in WM, no study has explored the nature of resource used in the FoA. Here, we explored this issue in a dual-task paradigm, in which we required participants to conduct a secondary task consuming distinct types of attention (object-based attention, spatial attention, and feature-based attention) during the maintenance phase of a retro-cue visual WM task. In seven experiments, we consistently found that only the secondary task consuming object-based attention impaired the retro-cue effect by lengthening the response time to the object in the FoA. These results suggest that FoA in WM requires object-based attention.

## The Effect of Rumination on Attentional Bias to Body Imagery

**Laura Dondzilo^1^, Petrina Wong^2^, Elizabeth Rieger^3^, Romina Palermo^1^, Sue Byrne^2^ and Jason Bell^2^**

^1^ARC Centre of Excellence in Cognition and its Disorders, School of Psychology, University of Western Australia, Australia; ^2^School of Psychology, University of Western Australia, Australia; ^3^Research School of Psychology, Australian National University, Australia

Attentional biases towards thin female bodies are associated with elevated levels of eating disorder symptomatology. However, the specific mechanism by which attentional biases trigger eating disorder symptoms remains unknown. One possibility is that a cognitive-affective process, rumination, plays a causal role in attentional bias. The current study investigated whether heightened rumination on eating, shape and weight concerns induced an attentional bias to thin female bodies, in young women. Participants were given a negative mood induction, followed by an induction of either a ruminative or distracting emotional regulation style. A visual dot probe task was used to measure attentional biases pre and post induction. Results compare the change in attentional bias pre and post and across groups to assess the causal role of rumination in attentional bias to body imagery.

The relationship between cognitive affective processes such as rumination and attentional biases to body imagery are then discussed.

## A Surround-Suppression Hypothesis for Attentional Guidance: Evidence From Visual Search

**Louis K. H. Chan**

Hong Kong Baptist University, Hong Kong

Most visual attention theories assume that visual saliency is defined by feature contrast levels. However, in common experience, no objects would look salient when a scene is full of contrasting items. The current study examined whether visual attention is guided by raw contrasts or some transformed signals. Participants searched for a unique-feature target in a display containing a cluster of homogeneous distractors and a cluster of heterogeneous distractors. Search was much more efficient when the target was among the homogeneous cluster. Since the target contrasted with all distractors regardless of its belonging cluster, this result reflected an attentional advantage for a visual item that was among low-contrast over high-contrast items. This suggests that visual saliency is a “contrast-of-contrast” signal, which could be formed when nearby objects suppress each other’s contrast level. A computer model that simulates the contrast calculations between visual items showed that surround suppression improves RT fit significantly.

## Implications of Visual Disorders

## Behavioural Measures of Cortical Hyperexcitability Assessed in People Who Experience Visual Snow

**Yu Man Chan^1^, Melissa Tien^2^, Lynette Millist^2^, Meaghan Clough^2,3^, Heather Mack^4^, Joanne Fielding^2,5^, Owen B. White^2^ and Allison McKendrick^1^**

^1^Department of Optometry and Vision Sciences, Faculty of Medicine, Dentistry and Health Sciences, The University of Melbourne, Australia; ^2^Department of Neurology, Royal Melbourne Hospital, Faculty of Medicine, Dentistry and Health Sciences, The University of Melbourne, Australia; ^3^School of Psychological Sciences, Monash Institute of Cognitive and Clinical Neurosciences, Monash University, Australia; ^4^Department of Ophthalmology, Royal Melbourne Hospital, Australia; ^5^School of Psychological Sciences, The University of Melbourne, Australia

Visual snow is a rare migraine-associated entity where people experience constant visual noise. One proposed mechanism is cortical hyperexcitability. We tested whether visual perceptual measures likely to be altered by excessive neural excitation, differed between 15 visual snow sufferers (aged 17–51) and 15 controls (aged 18–54). Four tasks were included: centre-surround contrast matching, luminance increment detection in noise, glass pattern coherence and global motion coherence thresholds. Neuronal architecture capable of encoding the luminance and contrast stimuli is present within primary visual cortex; whereas the extraction of global motion and form signals requires extrastriate processing.

The visual snow group demonstrated reduced centre-surround contrast suppression (*p* = .04) and elevated luminance increment thresholds for both high- (*p* = .01) and low-noise conditions (*p* = .04). Groups did not differ on the global form or motion task. Our data are consistent with cortical hyperexcitability and reduced suppression mechanisms in primary visual cortex in people who experience visual snow.

## Hyperosmotic Stress and Gene Adaptation in Myopia and Associated Ocular Diseases

**Sheila G. Crewther^1^, Nina Riddell^1^ and Alan Marshall^2^**

^1^School of Psychology & Public Health, La Trobe University, Melbourne, VIC, Australia; ^2^School of Life Sciences, La Trobe University, Melbourne, VIC, Australia

Myopia is the commonest visual disorder and also a high-risk factor for severe sight threating disorders. We have previously described morphological, ultrastructural and physiological changes associated with development of refractive errors. Here, biometric, elemental microanalysis (EDX) and RNAseq gene expression changes are related in the same model in the first 72 h of optical defocus. Refractive Compensation (RC) to +10D lenses is in equilibrium by 24 h while RC to −10D lenses continues for 72 h. RNA sequencing and Gene Set Enrichment Analysis demonstrate bi-directional gene network expression for structural, metabolic, and immune pathways while EDX shows that refractive compensation to both +10/−10D optical defocus is accompanied by hyperosmotic shifts in ion distribution profiles across the entire retina/choroid sclera within 24 h. Thus, hyperosmotic change in myopia needs to be considered as a risk factor for severe visual impairments while at the same time suggesting a path for the development of therapeutics

## Impaired Motion Salience and Disconnection of Ipsilateral FEF From the Attention Network in Strabismic Amblyopia

**Hao Wang^1^, Sheila Crewther^2^, Robin Laycock^2^, David Crewther^3^ and Zhengqin Yin^1^**

^1^Southwest Eye Hospital/Southwest Hospital, Key Laboratory of Visual Damage and Regeneration and Restoration of Chongqing, Third Military Medical University, Chongqing, China; ^2^School of Psychological Science, Faculty of Science, Technology and Engineering, La Trobe University, Melbourne, VIC, Australia; ^3^Centre for Human Psychophysiology, Swinburne University of Technology, VIC, Australia

**Purpose:** To compare performance and brain activation during motion and saccade tasks in adult strabismic amblyopes and controls.

**Methods:** fMRI activation and functional connectivity between parieto-frontal attention network ROIs, along with V1 and V5 were compared for the two tasks.

**Results:** Higher behavioural thresholds were required for amblyopic eye. Activation to motion and saccade tasks of amblyopic eye was weaker in IPS, FEF and V5 though no difference in V1 to motion task. Correlational connections of attention network of amblyopic eye were abnormal in motion task, with the FEF ipsilateral to the amblyopic eye the most isolated node. The network were normal for saccade task.

**Conclusions:** Amblyopic eye require slower speed for detection of moving targets. Specific deficit in functional connectivity between the FEF ipsilateral to strabismic eye and parietal attention network during motion task. Strabismic amblyopia is associated with reduced attention network activation during attention tasks independent of V1.

## Using Perceptual Learning to Mitigate Localized Blindness

**Paul Miller^1^, Guy Wallis^2^, Pete Bex^3^ and Derek H. Arnold^1^**

^1^School of Psychology, The University of Queensland, Australia; ^2^School of Human Movement, The University of Queensland, Australia; ^3^Department of Psychology, Northeastern University, Massachusetts, USA

Localised blindness can result from damage to the human visual system. There are no proven interventions to reduce the extent of blindness from neuropathology, but it has been suggested that rehabilitation might be achieved through training. We investigated this by examining the impact of visual training on blindness associated with the human blindspot.

– a region of insensitivity all people have in each eye due to the absence of photoreceptors at the optic disc. After 4 weeks of training, we found an ∼10% reduction in the extent of functional blindness associated with the trained blindspot. We have since begun preliminary investigations to see if this training protocol could benefit age-related macular degeneration.
– with promising results. Overall, our data suggest functionally defined blindness, including the human blindspot, can be reduced through training, and that effective protocols for this can be developed with normally sighted people and translated to benefit clinical conditions.

## The Effect of Stigma Towards Individuals With Mental Health Disorders on Social Perception

**Hannah Abdul Razak, Jeneva Ohan, Carmen Lawrence and Troy Visser**

School of Psychology, University of Western Australia, Australia

Mental health stigma and its impact on persons with schizophrenia contribute to a myriad of problems including social isolation. This study aims to understand the social impact of stigma towards persons with schizophrenia by examining its influence on eye gaze perception. Participants first read control and experimental vignettes depicting fictional characters without significant symptoms of a mental health disorder or with the symptoms and diagnosis of schizophrenia. Participants then completed a gaze-cueing task that required them to identify targets that were either spatially congruent or incongruent with the direction of the eye gaze of the face stimuli. Lastly, participants completed questionnaires assessing levels of stigma, including perceived social status and dangerousness towards each individual depicted in the vignettes. The results indicated that participants showed a significantly larger gaze cueing effect towards faces associated with schizophrenia. Moreover, magnitude of gaze cuing was positively related to perceived dangerousness and negatively related to perceived social status. The results suggest that stigmas about schizophrenia can significantly modulate social attention, and thus may form a foundation for social isolation experienced by individuals with the disorder.

## Symposia II: Representations of Face Identity and Emotion

## Detecting Emotional Expressions: Do Words Help?

**Emma Portch, Charity Brown, Russell Hunter and Jelena Havelka**

University of Leeds, UK

Access to emotion labels (e.g., sad) can aid our recognition of emotional facial expressions. In two experiments, we examine whether exposure to emotion words aids online detection of emotional expressions. Participants were briefly presented with a series of faces displaying sad or neutral expressions. Prior to each face exposure, participants repeated out loud an emotion or neutral word and when the face appeared detected whether it was emotional or neutral in expression. Emotion words were labels (“sad,” “surprise”) or related verbs (“sob,” “gasp”) and were relevant (“sad,” “sob”) or irrelevant (“surprise,” “gasp”) to the sad expressions on display. Preliminary findings indicate no advantage in detecting emotional expressions following exposure to relevant emotion words compared to irrelevant emotion words or neutral words. This was the case when relevant emotion labels (sad) and verbs (sob) were encountered. We consider how our findings contribute to understanding when and how language influences emotion perception.

## Facing a Difficult Task: Integrating Image Variation to Find Faces in Crowds

**James Dunn, Richard Kemp and David White**

University of New South Wales, Australia

People are often faced with the task of finding target faces in crowds. Theoretical accounts of visual search performance propose that this process relies on a search template containing a description of the target. Robust templates describe features that distinguish the target and make it stand out from the crowd. Two experiments were conducted to examine how to optimise faces search templates for visual search. We measured participant’s speed and accuracy when searching for familiar and unfamiliar faces in a crowd of 20 images, following three types of target exposure: (a) a single image of the target, (b) four different images of the target, or (c) a single image created by averaging 20 other images of the target. Average images improved the accuracy of target detection relative to single images. However, additional benefits were observed after exposure to multiple images, with improvements observed in both speed and accuracy. This benefit was observed for unfamiliar but not familiar faces, suggesting that exposure to variation compensates for an absence of familiarity.

## Similarity Asymmetries in Face Perception

**David White**

University of New South Wales, Australia

People recognise familiar faces despite substantial variations in facial appearance. The present work draws on a well-known phenomenon in the category learning literature “similarity asymmetry” to probe organisation of memory representations supporting this ability. Perceived similarity of category members in one sequence (A → B) differs from similarity in the opposite sequence (B → A). Because similarity asymmetries are systematically related to the relative prototypically of A and B, we predicted that averages of familiar faces and individual exemplars contributing to these averages would show asymmetries consistent with prototypical category structure. Our results show (a) systematic asymmetries consistent with average images occupying central regions of within-identity face space, (b) a strong relationship between relative familiarity of celebrities and the strength of asymmetries, (c) significantly smaller asymmetries in ratings made to unfamiliar faces. These results provide the first direct evidence for prototypical organisation of variation in representations of familiar faces.

## Attention: Neural and Perceptual Effects

## Measuring the Spotlight of Moving Attention

**Satoshi Shioiri^1^, Kei Ishii^2^, Kazumichi Matsumiya^1^ and Ichiro Kuriki^1^**

^1^Research Institute of Electrical Communication, Tohoku University, Japan; ^1^Graduate School of Information Sciences, Tohoku University, Japan

Moving objects can be tracked by visual attention independently of eye movements. To keep attending on a moving object, attention must be controlled dynamically. We developed a technique to measure spatiotemporal characteristics of visual attention while tracking a moving stimulus. The attentive tracking was performed with flickering disks, with which we measured attentional modulation at variable locations and its change in time by steady-state visual-evoked potential (SSVEP). Spatial modulation of attention estimated by SSVEP showed that attention effect peaked at a location ahead of the moving stimulus. This suggests that attention tends to be shifted in the direction of the moving object tracked.

Interestingly, we found that the P3 component of ERP showed the largest attentional effect at the location of the tracked stimulus. The difference between SSVEP and P3 may indicate different attention dynamics at different stages of visual processing.

## Signal Variability Undermines Confidence in Visual Perception

**Morgan Spence, Paul Dux and Derek Arnold**

School of Psychology, The University of Queensland, Australia

Humans intuitively evaluate their decisions by forming different levels of confidence. Although decisional confidence and sensitivity are highly correlated, recent evidence has shown that the range of direction signals within a global motion display disproportionately undermines confidence in global direction judgments, relative to precision. Here, we report that the range of different orientation signals in a global display also disproportionately undermines confidence in judgments of average display orientation. Performance was equated (∼70%) for stimuli containing different ranges of orientations uniformly distributed about a display average (±6°, 12°, 18° and 24°). Despite constant sensitivity, participants were less confident when judging the average orientation of more variable displays. These findings provide support for generalized computations underlying perceptual confidence that disproportionately weight the range of differently tuned cells responsive to an input.

## Spatial and Feature-Based Attention Have Different Effects on Population-Level Tuning

**Erin Goddard, Thomas A. Carlson and Alexandra Woolgar**

ARC Centre of Excellence in Cognition and its Disorders (CCD), Perception in Action Research Centre (PARC) and Department of Cognitive Science, Macquarie University, Australia

Spatial and feature-based attention can both change the stimulus related information signalled by single cells, but their effects on population-level coding is less well defined. We scanned participants (*n* = 10) using MEG, while they covertly attended to either an object on the left or the right (spatial attention manipulation) and reported the object’s shape or colour (feature-based attention manipulation). We used multivariate pattern classification analysis to infer the dissimilarity of the population response to attended and non-attended stimulus features, at attended and non-attended locations. We found that while both spatial and feature-based attention increased decodeability of object shape and colour, there were qualitatively different patterns of enhancement. Specifically, feature-based attention produced relatively greater enhancement of the smallest stimulus differences, while spatial attention produced greatest relative enhancement of intermediate stimulus differences. We describe these qualitatively distinct effects in terms of the normalisation model of attention (Reynolds & Heeger, 2009, *Neuron*, *61*(2), 168–185).

## Predictive Remapping Gives Rise to Environment-Centered Inhibition of Return

**Chuyao Yan^1^, Tao He^1^, Raymond M. Klein^2^ and Zhiguo Wang^3^**

^1^Department of Psychology, Hangzhou Normal University, Hangzhou, China; ^2^Department of Psychology and Neuroscience, Dalhousie University, Halifax, Canada; ^3^Department of Cognitive Science, Macquarie University, Sydney, Australia

Visual sampling is facilitated by inhibition of return (IOR), a mechanism that discourages attentional orienting towards previously inspected locations (or objects). Despite that saccadic eye movements constantly shift the retinal image of the world, IOR appears to operate in environment-centered coordinates. Here, we show that environment-centered IOR is supported by predictive remapping, a neural mechanism that has been suggested to maintain spatial constancy by predictively activating neurons to compensate retinal shifts caused by saccades. With a cueing task, Experiment 1 revealed an IOR effect at a retinal locus that had been brought to the cued location by a saccade. Experiment 2 further showed that, immediately before the initiation of a saccade, IOR was observed at a retinal locus that would be brought to the cued location by the same saccade. These results provide direct behavioral evidence that predictive remapping is a mechanism behind environment-centered IOR.

## Confidence Levels During Perceptual Decision-Making Are Discrete

**Andrei Gorea^1^, Matteo Lisi^2^ and Gianluigi Mongillo^3^**

^1^Laboratoire Psychologie de la Perception, Universite Paris Descartes & CNRS, France; ^2^Laboratoire Psychologie de la Perception, Universite Paris Descartes, France; ^3^Centre de Neurophysique, Physiologie et Pathologie, Universite Paris Descartes & CNRS, France

Are our decisions, and hence confidence therein, based on a full knowledge of the prior and likelihood probability functions? Each trial consisted of two consecutive decisions on whether a given signal was above or below some reference value. The first decision was to be made on a signal randomly drawn from a uniform distribution. Correct/incorrect responses resulted into signals randomly drawn from respectively the positive/negative sub-intervals to be judged when making the second decision. Subjects were told so. A non-Bayesian observer was designed to have discrete confidence levels instantiated by one-, two- or three-second decision criteria representing different levels of the point estimates of the evoked response. Synthetic datasets reliably discriminated Bayesian from non-Bayesian observers. Over nine subjects, the non-Bayesian observer with two–three confidence levels systematically outperformed the Bayesian observer in predicting the actual behavior.

Contrary to current claims, confidence appears to be a discrete rather than continuous quantity.

## Faces and Bodies II: Identity Recognition, Holistic Processing, and Attention

## Off With Their Heads: An Infant Body Inversion Effect With Headless Bodies

**Emma Axelsson and Chloe Kelly**

The Australian National University, Australia

Adults demonstrate face and body inversion effects (BIEs), that is, poorer discrimination of inverted compared to upright images. However, BIEs are reduced with headless bodies suggesting that heads are incorporated into adults’ body representations. Infants demonstrate face inversion effects at four months, but infants’ developing body representations are less clear. Seven-month-old infants’ fixations were measured, first to a single image, and then to the same image paired with an identical figure in a novel posture. Infants saw upright and inverted images, half with heads and half headless.

Infants’ novelty preferences to the novel postures were significantly greater than chance for the upright headless figures, but not the inverted. Novelty preferences for the upright and inverted whole figures were at chance. Infants looked longer at the heads than the bodies of the upright whole figures suggesting that heads detract attention from bodies possibly precluding infants’ capacity to discriminate bodies.

## Does Holistic Processing Impair Change Localization?

**Winnie Wing Lui Chan^1^, Sing Hang Cheung^1^ and William G. Hayward^2^**

^1^University of Hong Kong, Hong Kong; ^2^School of Psychology, The University of Auckland, New Zealand

Many people report the experience that it is easy to notice a change in someone’s facial appearance but hard to identify what the difference is. Some researchers have suggested that holistic processing of faces is sensitive to detecting changes but is poor in localizing changes (Wilford & Wells, 2010). We tried to validate this claim with featural and configural changes to composite faces, using both change detection and change localization tasks.

Aligned face composites interfered with change detection but did not affect change localization. There was no difference in performance between featural and configural changes. This result is consistent with the claim that holistic processing impairs change localization, but we wanted to extend this result using a different test of holistic processing, and so we ran another experiment testing participants with the two tasks with feature-changed whole faces or scrambled faces. We found an advantage for whole faces in both the change detection task and the change localization task. Thus, the localization impairment in holistic processing did not extend to this new paradigm. Although localization was impaired for composite faces, the second experiment shows that holistic processing of whole faces does not always reduce change localization ability.

## Unravelling Oculomotor Capture by Faces by Looking Through the Pupil’s Keyhole

**Christel Devue and Gina Grimshaw**

Victoria University of Wellington, New Zealand

Irrelevant faces capture the eyes through salient visual features; although, not systematically so. Here, we tested whether oculomotor capture is due to fluctuations in attentional preparation, as indexed by pre-trial pupil diameter. Participants performed a visual search task in which they made a direct saccade to a colour singleton presented among irrelevant objects and faces (angry or neutral). As expected, faces attracted the eyes more than other objects, regardless of facial expression. Pupil size reliably predicted saccade accuracy. However, comparably small pupil diameters preceded both accurate saccades and oculomotor capture by faces. Small pupil diameters may thus index neural preparation to detect salient events, irrespective of their task-relevance. Capture saccades (and other errors) were faster than correct saccades, reflecting reduced oculomotor control. Our findings thus suggest that oculomotor capture brings faces into foveal vision to decode potentially important meaning (emotion, identity) and is promoted by distributed attention and goal flexibility.

## Enhancing CCTV: Averages Improve Human and Computer Face Identification From Poor-Quality Images

**Kay L. Ritchie, Robin S. S. Kramer, Eilidh Noyes, Rob Jenkins and A. Mike Burton**

Department of Psychology, University of York, UK

Low-quality CCTV images are problematic for forensic face recognition. Previous studies have shown that face averages comprising multiple images of the same person improve computer recognition. Here, we test the advantage of averages with pixelated images in human and computer face identification. Using multiple images of each identity, we created sets of unpixelated and pixelated images and their averages. Experiment 1 tested human face matching and found no average advantage for unpixelated images, but a decrease in accuracy for pixelated images which was rectified by averaging those images together.

Experiments 2 and 3 tested computer face recognition using linear discriminant analysis (LDA) and a commercially available face recognition app respectively. Both experiments showed the same pattern of results, with poorer recognition of pixelated images and a significant improvement with their averages. These results have implications for automatic face recognition, particularly in identifying faces from poor-quality CCTV images.

## Poor Recognition of Other-Race Faces Cannot Be Explained by a Lack of Motivation

**Kate Crookes and Gillian Rhodes**

ARC Centre of Excellence in Cognition and Its Disorders, School of Psychology, University of Western Australia, Australia

People are generally better at recognising own-race than other-race faces. This “other-race effect” is very well established, yet the underlying causes are still debated. One socio-cognitive explanation is that the effect stems from a lack of motivation to individuate other-race faces. The present study investigated participants’ motivation and meta-cognition regarding own-race and other-race face recognition. Caucasian participants completed the Australian and Chinese versions of the Cambridge Face Memory Task, once with the standard timing (i.e., 3 s per study face) and once with unlimited, self-paced study phases. Contrary to the motivational account participants in the self-paced condition did not spend longer studying own-race than other-race faces nor was their other-race effect increased. Moreover participants reported applying significantly more effort to recognising Asian than Caucasian faces with no participants reporting spending greater effort on Caucasian faces. These results provide no support for the socio-cognitive motivational explanation of the other-race effect.

## Coding Facial Identity: Evidence for a Channel Tuned to the Average (Norm) Face

**Linda Jeffery^1^, Nichola Burton^1^, Stephen Pond^1^, Colin Clifford^2^ and Gillian Rhodes^1^**

^1^ARC Centre of Excellence in Cognition and its Disorders, School of Psychology, The University of Western Australia, Australia; ^2^The University of New South Wales, Australia

Face identity may be coded relative to an average face that serves as a perceptual norm. We tested this norm-based coding account and asked how the norm is coded. We used two aftereffect paradigms to rule out a no-norm narrow-band multichannel model and to distinguish between a two-channel model, with the norm represented by equal activation in oppositely tuned channels, and a three-channel model, with an additional channel tuned to the norm. Experiment 1 ruled out narrow-band multichannel coding because aftereffects increased sharply and then plateaued as adaptors become more extreme. Experiment 2 ruled out the two-channel model because adapting to alternating images from opposite ends of an identity trajectory widened the range of faces identified as the average whereas adapting to the centre (average) narrowed the range. These opposite shifts are not possible in the two-channel model, but are expected on the three-channel model.

## Colour Vision

## How to Design Good Colour Maps

**Peter Kovesi**

Centre for Exploration Targeting, School of Earth and Environment, The University of Western Australia, Australia

Many colour maps used for rendering pseudo-colour images have highly uneven perceptual contrast. It is not uncommon for colour maps to have perceptual flat spots that can hide a feature as large as one tenth of the total data range. Colour maps may also have perceptual discontinuities that induce the appearance of false features. Previous work in the design of perceptually uniform colour maps has mostly failed to recognise that CIELAB space is only designed to be perceptually uniform at very low-spatial frequencies. In order to resolve fine structures in an image, the most important requirement of a colour map is that the magnitude of the incremental change in perceptual lightness of the colours is uniform. This paper presents specific design requirements for linear, diverging, rainbow and cyclic colour maps. To support this work, two test images for evaluating colour maps are also presented.

## Phototransduction Genes in Vertebrate Vision: Evolutionary Origin of Rod and Cone Isoforms

**David M. Hunt^1^, Aaron Chuah^2^, Hardip Patel^2^, Wayne I. L. Davies^1^, Nathan S. Hart^3^, Shaun P. Collin^4^ and Trevor D. Lamb^5^**

^1^School of Animal Biology and Lions Eye Institute, The University of Western Australia, Australia; ^2^Genome Discovery Unit, ACRF Biomolecular Resource Facility, Australian National University, Australia; ^3^Department of Biological Sciences, Macquarie University, Australia; ^4^School of Animal Biology, Lions Eye Institute, Oceans Institute, University of Western Australia, Australia; ^5^Department of Neuroscience and ARC Centre of Excellence in Vision Science, John Curtin School of Medical Research, Australian National University, Australia

In vertebrates, rod photoreceptors are responsible for vision in dim light and cone photoreceptors for vision in daylight, and for colour vision. Following photon capture, the signal is amplified through the sequential steps of phototransduction which are essentially the same in rods and cones. Surprisingly, however, the different components of the phototransduction process are largely driven by rod- and cone-specific isoforms. These isoforms most likely arose by gene duplication but in many cases, it is uncertain when this occurred in vertebrate evolution.

To address this, we have de novo assembled eye/retina transcriptomes generated by RNA-Seq from 14 ancestral species that are representative of the jawless (agnathan) and jawed (gnathostome) vertebrate lineages. The presence of isoforms was assessed by sequence homology and phylogenetic analysis. Considerable variation is evident between species in the presence of isoforms but is generally consistent with an origin from early whole genome duplication events.

## Asymmetric Single-Pulse Detection Thresholds and Temporal Double-Pulse Discrimination Thresholds of S-Cone Pathways

**Lin Shi**

Kunming University of Science and Technology, China

The S-cone pathway showed different temporal characteristics on increase and decrease intensity stimulation of S-cone and various S-cone adaptions. My previous research showed that temporal resolutions at two S-cone adaption conditions were different (i-Perception Vol. 5–4, p. 218). Here, I studied the temporal resolution of the S-cone pathways in details.

First, the single-pulse detection thresholds were measured from 12 normal color vision observers using a Gaussian patch which chromatic coordinates were gradually changed from the peak to the background alone positive and negative directions of the S-cone axis in a physiologically relevant LMS space. The stimuli were presented by a calibrated visual stimuli system which display error rate of the LMS coordinates inside the gamut of a 10-bit-driven CRT was less that 5%. The LMS coordinates of the background were sampled from 15 points located at the S/LM and the L/LM axes at following levels, S/LM: 0.8, 0.9, 1, 2, 3, L/LM: 0.3, 0.335, 0.37. Second, the double-pulse ISI discrimination thresholds were measured at the intensities of previous single-pulse detection thresholds. Results showed that both the single-pulse detection thresholds and the temporal double-pulse discrimination thresholds were different between positive and negative directions of S/LM axis. The results indicated asymmetric S-cone pathways on aspects of both sensitivities and temporal resolutions.

Acknowledgment: This study supported by National Natural Science Foundation of China (61368005).

## New Findings Into the Loss of Colour Vision in Retinal Disease

**John L. Barbur**

Applied Vision Research Centre, School of Health Sciences, City University London, UK

Advances in colour assessment techniques yield reduced within subject variability and this leads to more accurate isolation and assessment of both red/green and yellow/blue chromatic mechanisms. These techniques have recently been applied to study the effects of normal aging on colour vision and to establish reliable, upper, normal age limits. Many aspects of congenital colour deficiency including the presence of anomalous dichromatic colour matches can now be understood as a result of significant advances in the genetics of colour vision. Acquired loss of chromatic sensitivity continues to remain a challenge, particularly when the subjects also have congenital colour deficiency. New findings that describe the type and extent of colour vision loss in diseases of the retina such as age-related macular degeneration will be presented. The loss of colour vision in subjects with systemic diseases such as diabetes, in the absence of retinopathy, will also be described.

## Cortical Representation of Color Categories in Prelingual Infants

**Ichiro Kuriki^1^, Jiale Yang^2^, So Kanazawa^3^ and Masami K. Yamaguchi^2^**

^1^Tohoku University, Japan; ^2^Chuo University, Japan; ^3^Japan Women’s University, Japan

A close relationship between colour categories and language is often considered as a strong support for the Sapir-Whorf hypothesis. However, it is controversial whether colour categories are defined only through the language acquisition process. To address to this issue, we measured brain-activity changes in prelinguistic infants (5 to 7 months old) by using near-infrared spectroscopy (NIRS). Two types of visual stimuli were used: colour alternations (1 Hz) between green and blue or two shades of green, while colour differences were equated in CIE Lab space. Channels around both left and right occipito-temporal cortex showed selective increase in oxy-Hb for the between-category stimulus, but not in occipital region. Among 12 NIRS channels a significant activity was observed in the posterior part in both hemispheres. The adults’ NIRS responses also showed significant increase to between-category stimulus, but no significant laterality. Our results suggest that categorical colour perception could be initiated by visual, non-lingual, processing.

## Action, Binocular vision, Colour, Brightness, and Motion

## The Profile of Attention Changes Across Locations Relative to Reach Direction

**Anna Ma-Wyatt and Emma E. M. Stewart**

School of Psychology, University of Adelaide, Australia

Attention shifts to the goal of an eye or hand movement. Perisaccadic mislocalisation can differ dependent on the location of a probe relative to saccade direction (Kaiser & Lappe, 2004). While links have been made between saccadic compression and the pre-motor attentional shift, it is not known if the spatiotemporal profile of attention changes at different locations relative to the direction of the movement. We measured performance at locations orthogonal or parallel to the movement direction for reach only, reach plus saccade and saccade only. We found that the spatiotemporal profile of attention differs across different movement combinations, and is also different at different locations relative to movement direction. These results suggest that attentional guidance may be more important at differing timepoints, depending on the type of movement being enacted.

## Investigation of the Impacts of Exercise Experience on Cognitive Functions

**Yi-Min Tien^1^, Ting Tzu Chang^1^, Chia-Ying Lee^1^ and Li-Chuan Hsu^2^**

^1^Department of Psychology, Chung Shan Medical University, Taiwan; ^2^School of Medicine, China Medical University, Taiwan

It is widely accepted that regular exercise benefits both physical and mental health. While it is unclear whether sport expertise impacts cognitive functions. Current study adopted two visually presented tasks, including choice reaction task (CRT) and stop-signal task (SST), to measure participants’ response speed, accuracy, and inhibition function. Seventy-six college athletes and 31 university students were recruited as expert group and control group respectively. The results show that, in CRT, expert group shows significantly shorter reaction time than control group while no difference was found in accuracy. In SST, expert group shows significantly shorter stop-signal reaction time than control group while no difference was found for Go reaction time and accuracy. The results suggest that the sport expertise promotes performance of athletes not only in relatively simple response speed but also in executive inhibit function.

## Effects of Task Difficulty on Postural Control in Dart Throwing

**Yong-Hyun Lim, Emese Foldesi, Ju-Hun Kim and Nam-Gyoon Kim**

Department of Psychology, Keimyung University, South Korea

We investigated postural coordination in dart throwing. Darts can be thrown using only the elbow and wrist while keeping the rest of the body stationary. In order to introduce variability in the coordination pattern, sizes of the target (Experiment 1), distances to the target (Experiment 2), and the characteristics of support surface (Experiment 3) were varied. Dart throwing data were obtained using a wireless motion tracking system via sensors attached to the index finger, wrist, elbow, shoulder, hip, knee, and ankle of the right side (the throwing hand) with additional sensors attached to the head and the left shoulder, for a total of nine sensors. Cross-correlations between joints (wrist-elbow, wrist-shoulder, wrist-hip, wrist-knee, elbow-shoulder, elbow-hip, elbow-knee, shoulder-hip, and shoulder-knee) were used to construct coordination patterns. The standard deviations of the head and the right shoulder motion were used to assess body sway. In each condition of target size (Experiment 1), distance (Experiment 2) and support surface (Experiment 3), participants threw darts 20 times, preceded by 20 practice throws. Different patterns of coordination arose as a function of target size, target distance, and support surface. Different body segments were required to complete the task of throwing darts under different task constraints. Effects of target size and target distance on body sway were minimal. Of particular interest was the finding that body sway was less in the narrow beam condition than in the wide plank or foam-rubber mattress conditions. Results suggest that the posture system is integrated with the task of dart throwing, facilitating the task under different constraints.

## Retinal Independent Depth Aftereffects of Stereoscopic Textural Surfaces

**Shufang He and Hiroaki Shigemasu**

Kochi University of Technology, Japan

Although several studies showed depth aftereffects independent of retinal position and suggested the adaptation of higher order depth processes, the adaptation of three-dimensional textural surfaces has not been investigated. We used stereoscopic dynamic random-dot plaid patterns consisting of two orthogonal oriented sinusoidal depth gratings to create lumpy surfaces. After adapting to two horizontally positioned plaid stimuli with larger and smaller amplitude of sinusoidal waves than test stimuli, observers were asked to judge which side of the test stimuli appeared to have the larger amplitude. The amplitudes of test stimuli were manipulated randomly in each trial and point of subjective equality (PSE) was calculated. To examine the independence of local adaptations to the lumps, the phase position was randomly changed during the adaptation and the PSE was compared with static phase and no-adapter conditions. Results showed similar degree of negative aftereffects with both random and static phase adapters and suggest the higher order adaptation to three-dimensional textural surfaces.

## Olfactory Effects on Visual Perception for Pictures and Words in Binocular Rivalry

**Maiko Komura, Maiko Uesaki, Kiyofumi Miyoshi and Hiroshi Ashida**

Department of Psychology, Graduate school of Letters, Kyoto University, Japan

Zhou et al. (2010) showed that olfactory information modulates visual perception in binocular rivalry. Olfactory stimuli prolong perception of congruent visual stimuli. However, little is known how olfaction affects visual processing in more details. We investigated how the effects of olfaction on binocular rivalry differ by the types of visual stimuli. In Experiment 1, we examined whether the olfactory effects differ for visual stimuli of pictures or words (Chinese characters). The effect of olfaction was found for pictures, but not for words. In Experiment 2, we used words (katakana) in colors that were either congruent or incongruent with its meaning. The effect of olfaction was found on colors, but not on words. These results suggest that the effects of olfaction differ by the types of visual stimuli, and the differences may be caused by the familiarity of visual stimuli or the strength of association between olfaction and visual objects.

## The Influence of Phase-Alignment for Interocular Suppression in Natural Scenes

**Yu-Min Tai and Pi-Chun Huang**

Department of Psychology, National Cheng-Kung University, Tainan, Taiwan

We used natural scenes as stimuli instead of simple periodical visual patterns to probe the effects of phase-alignment information to interocular suppression. The pattern masking paradigm was used to measure the discrimination threshold of natural scene images that were band-pass filtered. Two types of pedestal were used. One was identical to the target (blur pedestal) and the other was the original image (non-blur pedestal). The thresholds were compared under monocular, binocular, and dichoptic viewing conditions. The results showed typical dipper functions under monocular and binocular viewing conditions for both blur and non-blur pedestals. The dichoptic pedestal caused a much stronger masking effect. However, non-blur pedestal produced a weaker masking effect across all the pedestal contrasts, and the masking strength between these two pedestals did not change under the three viewing conditions. The results support the importance of phase-alignment in pattern masking, and the influence occurred before the binocular summation stage.

Acknowledgement: This work was supported by MOST 104-2628-H-006-001-MY3 (to P. C. H.).

## Disparity Fusion Limits Are Unaffected by Interocular Blur Differences

**Philip M. Grove, Alanna Koh and Ashleigh L. Harrold**

School of Psychology, The University of Queensland, Australia

Binocular images can differ in blur level due to, for example, pathology, or due to downsampling one image to economize on bandwidth in 3D television broadcasts. Some effects of binocular blur asymmetries are known. For example, blurring one eye’s input degrades stereoacuity. However, it is not known how blurring one eye’s image affects disparity fusion limits. We measured disparity fusion limits for target features in dichoptic images, one sharp and one blurred, of real scenes depicting a range of depth intervals at each of four blur levels. We determined the fusion limit using the method of constant stimuli and defined the limit as the depth interval for which diplopia was reported on 50% of the trials. Disparity fusion limits were approximately constant across all blur levels. Based on this experiment and a control experiment, we conclude that disparity fusion limits are determined by the sharpest image available to either eye.

## Binocular Fusion Limits Depend on Eccentricity, Not Stimulus Separation

**Ashleigh L. Harrold and Philip M. Grove**

School of Psychology, The University of Queensland, Australia

There is general agreement that disparity fusion limits increase with eccentricity. However, Howard and Rogers (2012), note that the reported increase of fusion limits with eccentricity is confounded with separation between the test stimulus and the fixation point. To address this confound, we adapted the procedures used by Levi and Klein (1990) to measure stereoscopic fusion limits. We created three iso-eccentricity circles centred upon the fixation point with radii of 0.73°, 2.19° and 5.06°. The test and reference stimuli were presented on this circle and separation was manipulated by moving the test stimuli away from the reference along each circle. Using the method of constant stimuli, we computed the 75% diplopia threshold for crossed and uncrossed disparities as the fusion limit at each location. Fusion limits increase with eccentricity but less so with separation, indicating that the increase in fusion limits is almost exclusively due to an increase in eccentricity.

## Anisotropy in Visual Texture Recognition of Natural Pattern

**Toshihiro Bando^1^ and Yasunari Sasaki^2^**

^1^Doshisha Unversity, Japan; ^2^Kanazawa Seiryo University Women’s Junior College, Japan

We perceive surface feature of object with vision as well as tactile sense. In our study, we find out spatial anisotropy in visual texture recognition of natural pattern. That is, there are some cases that perceived textural feature of the image is distinctly changed only by the rotation of the same image. The reason why this perceived change of texture occurred seems to be that the gradation pattern on the surface of the object regarded as the shadowgram caused by fine asperity of object surface and illumination. Relative position change of gradation pattern by the image rotation caused context change and our interpretation of the pattern distinctly changed. In other words, context change of shade pattern leads to change of texture recognition. On the other hand, rotation of the texture pattern does not always lead to distinct change of texture. Ecological perspective seems to be important to understand this phenomenon.

## Effects of Adding a Color Stimulus Sequence to a Spatial Response Sequence on Visuomotor Sequence Learning

**Kanji Tanaka, Takashi Kawai and Katsumi Watanabe**

Waseda University, Japan

Here, we investigated the effects of adding a color stimulus sequence to a spatial response sequence on explicit sequence learning. The participants learned a sequence of spatial button presses by trial-and-error process. First, all participants learned a non-colored spatial sequence, in which the response button always turned red. In the subsequent task, the participants were assigned to a colored or a non-colored sequence group. In the colored sequence group, the spatial response buttons turned pre-determined colors. The results showed that the colored sequence group acquired the correct button presses of the sequence earlier, but showed a slower mean performance time than the non-colored sequence group. These results indicate that if participants explicitly attend to both a spatial and color stimulus sequence simultaneously, it helps learning the sequence more quickly but the automatization of motor responses is hindered to some extent, presumably because additional process required for color sequences.

## Effects of 3D shape on Lightness Perception

**Keiji Kato, Keita Hirai and Takahiko Horiuchi**

Graduate School of Advanced Integration Science, Chiba University, Japan

Color appearance models are important for color management in product designs. Due to the development of 3D printing technologies, a color appearance model for 3D objects is recently required. In this paper, as an early-stage experiment, we investigated effects of displayed 3D shapes on lightness perception. In our subjective experiments, a 3D shape image and a color patch were presented side-by-side on a calibrated color display.

Observers evaluated their perceived lightness to the 3D shape by adjusting lightness of the color patch via a computer. We prepared 140 texture stimuli in total generated by the combination of seven colors (dark gray, neutral gray, light gray, and four colors), five objects with different shapes and four lighting directions. Our results suggest that perceived lightness to 3D shape images corresponds to approximately 70% of the maximum lightness in each image. In addition, lighting conditions significantly influence on perceived lightness.

## Effects of Texture on Lightness Perception

**Keiji Kato, Keita Hirai and Takahiko Horiuchi**

Graduate School of Advanced Integration Science, Chiba University, Japan

Color appearance models are important for color management in product designs. Recently, in the field of digital fabrication technologies, a color appearance model for texture surfaces is required. In this study, as an early-stage experiment, we investigated effects of textures on lightness perception. In our subjective experiments, a texture stimulus and a color patch were presented side-by-side on a calibrated color display. Observers evaluated their perceived lightness to the texture by adjusting lightness of the color patch via a computer.

We prepared 161 texture stimuli in total generated by the combination of seven colors (dark gray, neutral gray, light gray, and four colors with neutral gray) and 23 textures. Our results suggest that perceived lightness to neutral gray textures is almost the same as that to color patches. In contrast, perceived lightness to light gray textures is significantly lower than that to color patches.

## Influence of Viewing Conditions on Perceptual Qualities of Materials

**Keiji Kato, Keita Hirai and Takahiko Horiuchi**

Graduate School of Advanced Integration Science, Chiba University, Japan

Color appearance of objects is influenced by viewing environments such as surrounding illumination, viewing angle, and brightness. Then color appearance models have been developed based on the appearance of color patches. However, influences of viewing environments on texture perception have not been investigated. In this paper, we analyzed texture perception of materials under various viewing conditions. Our visual experiments were conducted based on perceptual qualities defined by Fleming et al. (2013). Observers evaluated the perceptual qualities under the following five viewing conditions: (a) standard, (b) reducing display luminance, (c) changing display color temperature, (d) far viewing distance, and (e) brighter surrounds. Our results showed the following findings: (a) The perceptual quality “Hardness” is increased by reducing display luminance and brightening surrounds of presented materials, (b) “Roughness” and “Fragility” are significantly decreased when viewing distances are farther, and (c) “Coldness” is decreased and “Prettiness” is increased by reducing display color temperatures.

## Contribution of ipRGC to the Brightness Perception

**Masahiko Yamakawa^1^, Sei-ichi Tsujimura^2^ and Katsunori Okajima^1^**

^1^Yokohama National University, Japan; ^2^Kagoshima University, Japan

ipRGCs, the photoreceptors expressing the photopigment melanopsin, are known to have particular functions, such as circadian rhythm entrainment and pupil light reflex.

However, it is still unclear how ipRGCs contribute to the visual perception. We investigated how the ipRGCs are involved in the brightness perception to elucidate the contribution to the image forming pathway. We used a six-primary projector system so that the effect of ipRGCs on brightness perception can be isolated from that of cones and rods with a silent substitution method. In addition, pupil diameter was measured under each light stimulus to evaluate the actual effects of ipRGCs and cones. The results showed that the magnitude of brightness can be explained by a formula as functions of cone and ipRGC responses quantitatively. The derived formula for brightness perception suggests that ipRGCs modify the gain of luminance signals on the brightness.

## Adaptation to Velocity Profiles of Random Moving Dots: A Motion “Smoothness” Aftereffect

**Jit Wei Ang and Gerrit Maus**

Nanyang Technological University, Singapore

Previous reports of motion adaptation range from low-level adaptation of speed and direction to high-level adaptation of biological motion. However, little is known about potential mid-level processes that could contribute to higher level aftereffects. Using random dot kinematograms, we adapted observers to two velocity profiles with matched average speed: sinusoidal variations in velocity (“smooth”) or constant speed with abrupt direction reversals (“jerky”). Subsequently, we tested perception of different velocity profiles that ranged from smooth to jerky by either morphing the smooth and jerky profiles with a “neutral” (Gaussian) profile, or by varying the ratio of smooth and jerky dots. In a 2AFC task, observers judged which of two differently adapted stimuli looked smoother. All observers judged subsequent stimuli as “jerkier” after adapting to “smooth” motion and vice versa. Adaptation to different velocity profiles is unlikely to depend on lower level adaptation, but might contribute to higher level aftereffects, as in biological motion.

## Older Adults Integrate Motion Direction Over Larger Areas for the Perception of Small Moving Stimuli

**Thomas J. McDougall^1^, Bao N. Nguyen^2^, Allison M. McKendrick^2^ and David R. Badcock^1^**

^1^School of Psychology, The University of Western Australia, Australia; ^2^Department of Optometry and Vision Sciences, The University of Melbourne, Victoria, Australia

Older observers demonstrate decreased behavioural surround suppression for motion direction discrimination, possibly due to decreased cortical inhibition in the aging brain. This implies the extent of summation is greater in older observers since weaker inhibition would allow the excitatory receptive field to be measureable over larger areas. Here, we investigated the effects of aging on spatial summation of motion direction using the Battenberg summation method which circumvents internal noise changes by holding overall display size constant, producing more accurate estimates of summation area. In this checkerboard stimulus, check size (luminance-modulated drifting gratings) is varied to measure dependence on signal area. Contrast detection thresholds for motion direction discrimination of these patterns were measured in 14 younger (24–34 years) and 14 older (62–76 years) observers. The magnitude of spatial summation differed between groups for the smaller checks, suggesting that older observers integrate over larger areas when processing small moving stimuli.

## The Effect of Congruent Directional Sound on the Sampling Efficiency of Motion Direction Discrimination Performance

**Ang-Ke Ku and Pi-Chun Huang**

Department of Psychology, National Cheng-Kung University, Tainan, Taiwan

It has been shown that congruent sound could improve motion direction discrimination. We used an equivalent noise (EN) paradigm to clarify whether the improvement was due to change of internal noise or sampling efficiency. The visual stimuli consisted of 100 dots in which the moving directions were sampled from a normal distribution with various standard deviations (SDs). The mean direction in order for the observers to determine differences from vertical-upward motion under different SDs and sound conditions (absent, stationary, incongruent, and congruent sound) were compared. Our results revealed the thresholds increased with SD, but the increasing rate differed along sound conditions. Our fits with an EN model showed that sampling efficiency was highest under the congruent sound condition and decreased under stationary and incongruent conditions without changing the internal noise level. In conclusion, congruent sound did not decrease internal noise but increased sampling efficiency in motion direction discrimination.

Acknowledgement: This work was supported by MOST 104-2628-H-006-001-MY3 (to P. C. H.).

## The Interactions Between Orientation and Motion Direction in A Global Coherence Task

**Pi-Chun Huang**

Department of Psychology, National Cheng Kung University, Tainan, Taiwan

In this study, an investigation of how a global percept is formed when local features have inconsistent image statistics was conducted. The stimuli were an array of moving Gabor elements whose orientation and moving direction were sampled from a normal distribution with a fixed variance. The mean motion direction was either orthogonal or parallel to the mean orientation. In different trials, either the mean orientation discrimination threshold from the vertical or the mean direction threshold from an upward motion direction was measured. It was found that the discrimination threshold was lower when the mean motion direction and orientation were orthogonal to each other. However, the discrimination threshold was invariant with the correlation between the sampling distributions for orientation and motion direction. The results suggested that while the visual system is sensitive to the mean of the image statistics, it ignores the correlation between orientation and motion direction.

## Differential Representation of a Visual Self-Motion Signal in Human Medial Optic-Flow Regions Revealed by Multi-Voxel Pattern Analysis

**Atsushi Wada, Yuichi Sakano and Hiroshi Ando**

Center for Information and Neural Networks (CiNet), National Institute of Information and Communications Technology, Japan

Visual optic-flow is important for estimating self-motion. The current fMRI study revealed differential univariate and multi-voxel response properties to visual self-motion signals among the following optic-flow regions recently identified in human medial cortex: visual area V6, the precuneus motion area (PcM), and the cingulate sulcus visual area (CSv). Along with optic-flow selective univariate activation in these medial regions, we found a unique inhibitory effect of self-motion incompatible signals in the CSv. Furthermore, our multi-voxel pattern analysis (MVPA) results identified a visual self-motion specific information in the PcM and CSv, but not in V6. Conversely, stimulus size had a strong, partial, and absent effect on the multi-voxel pattern in V6, the PcM, and the CSv, respectively, which may reflect the known retinotopic representation in V6 and the absence of such organization in the CSv.

These results suggest the importance of the medial optic-flow regions, especially the CSv, in visual self-motion processing.

## Colour and Binocular Cues

## Advantages of Colour Signals in Visual Search in Subjects With Congenital Deficiency

**Marisa Rodriguez-Carmona, Joseph Hickey and John Barbur**

Applied Vision Research Centre, School of Health Sciences, City University London, UK

Colour is arguably an effective and compelling, but also attractive and efficient method to enhance visual performance. We assessed how severity of colour vision loss in subjects with congenital deficiency relates to the loss of visual performance in colour-related tasks. A new test was designed specifically to quantify how colour signals affect the time needed to complete visual search tasks in large visual fields as well as the percentage correct scores when using stimuli that are typical of those employed in air traffic displays. In addition to confirming known results, this study reveals the importance of ∼effective” contrast of coloured stimuli (particularly in protan- and deutan-like subjects) and the significant advantages of yellow-blue colour signals in large displays. Equally importantly, the use of suprathreshold, “pastel” colours (with large yellow-blue components) enable mild colour deficient subject to carry our large-field display tasks with the same accuracy and speed as normal trichromats.

## History and Theory of Simultaneous Colour Contrast

**Robert P. O’Shea^1^, Nicholas J. Wade^2^, Stefano Brini^3^ and Urte Roeber^4^**

^1^School of Psychology and Exercise Science, Murdoch University, Australia; Discipline of Psychology, School of Health and Human Sciences, Southern Cross University, Coffs Harbour, Australia,; ^2^Department of Psychology, University of Dundee, UK; ^3^School of Psychology and Exercise Science, Murdoch University, Australia; Department of Psychology and Speech-Language Pathology, University of Turku, Finland; ^4^School of Psychology and Exercise Science, Murdoch University, Australia

Simultaneous colour contrast describes differences in colour of a patch from different surround colours. For example, a grey patch surrounded by black appears grey but when surrounded by red appears tinted with cyan. General descriptions of the phenomenon were made by Aristotle, Ibn al-Haytham, and Leonardo da Vinci, but more detailed studies were reported by Goethe (1810), Chevreul (1839), and Ragona-Scinà (1847). We report that some of the key observations of simultaneous colour contrast were made earlier than usually recognized:

– von Guericke (1672) described coloured shadows more than a century prior to Goethe.
– Monge (1789) described simultaneous colour contrast 50 years prior to Chevreul.
– Osann (1833) described a popular method for seeing simultaneous colour contrast 14 years prior to Ragona-Scinà.

We argue that simultaneous colour contrast remains unexplained, presenting a challenge to any comprehensive theory of colour vision.

## Sources of Monocular Depth Cues in Ternary Texture of Constrained Correlation Order

**Jessica Herrington^1^, John W. G. Seamons^1^, Marconi S. Barbosa^1^, Jonathan D. Victor^2^ and Ted Maddess^1^**

^1^Department of Neuroscience, John Curtin School of Medical Research, Australian National University, Australia; ^2^Department of Neurology & Neuroscience, Weill Cornell Medical College, NY, USA

The human visual system must minimize informational redundancy whilst maintaining behaviorally relevant information. We explored binary (black and white) textures with correlations constrained at orders 1 to 4. We explored 390 texture types using crowdsourcing platform Amazon Turk. Some ternary (black, white, grey) textures exhibited unexpected monocular depth cues. This abstract also reports preliminary results regarding those cues.

The study was done in two Turk experiments with 532 and 427 participants. Textures were presented in a 4AFC. Iso-discrimination levels were determined from fits to Weibull functions. For monocular depth cues, ternary textures were presented on a mean grey background, and subjects rated textures on a 5-point depth rating scale.

For the monocular depth cue study, texture split into depth planes was biased to fourth-order correlations while being as random as possible for lower orders. Future experiments will examine whether correlation order defines these monocular depth cues.

## Event-Related Brain Potential (ERP) Signatures of (Un)conscious Visual Perception Using Binocular Rivalry

**Urte Roeber**

School of Psychology and Exercise Science, Murdoch University, Perth, Australia

I will review experiments investigating the neural processing of stimuli that are, or will not be, perceived. To control perception, I used binocular rivalry. It occurs when each of the two eyes is presented with a different image, yielding alternating visibility of one image and invisibility of the other. I measured neural processing using event-related potentials (ERPs) from electroencephalographic recordings. Using a variety of experimental paradigms, my colleagues and I found:

– The earliest neural correlate of consciousness at about 100 ms after a change in stimulation whether this change was visible or not.
– The timing of the earliest neural correlate of consciousness to depend on the stimulus property that yielded the (in)visible change: shortest for orientation, longer for colour, and even longer for motion.
– The ERP amplitude to predict a change in the contents of visual consciousness by about 1000 ms.

## Eye Movements

## The Effects of Alcohol on the Kinematics of Reaching and Grasping

**Brian Timney, Steven Matson, Devon Silverberg and Melvyn Goodale**

Western University, Canada

Recent fMRI evidence suggests that alcohol reduces effective connectivity in cortical areas that are responsible for visuomotor coordination. We investigated the effects of moderate blood alcohol levels on the kinematics of reaching and grasping in order to assess the behavioural consequences. Participants were asked to reach for and pick up square blocks of different sizes placed at different distances. They did this while sober and with a blood alcohol level of approximately .08%. IREDs mounted on their wrists and fingers allowed us to monitor reaches using an OptoTrack motion sensing system. The data showed that, on average, reaches tended to be slower overall and took longer to reach peak velocity for all block sizes and distances. For maximum grip aperture, the pattern of results was less consistent. The results suggest that alcohol might affect reach and grasp components in different ways.

## Variability in V1 Activity and the Latency of Visually Guided Saccades

**Kayeon Kim and Choongkil Lee**

Seoul National University, South Korea

The latency of visually guided saccadic eye movements shows a surprisingly large variability even in identical stimulus condition. The variability correlates with the neural activity in diverse brain structures as early as V1. The significant correlation between V1 activity and saccade latency may indicate a link involving a functional role of V1, or a spurious link involving a confounding source such as attentional modulation of both V1 activity and saccade generation in progress of experimental session, thus non-functional role of V1. We summarize the results on the V1 activity before and after saccade target onset that relates to saccade latency and occurrence of express saccades, and on the changes in the V1 activity and saccade latency in progress of session that discount the spurious link. The results support the idea that V1 activity can predict the latency of upcoming visually guided saccades and occurrence of express saccades in identical stimulus conditions.

## Adaptation of Gaze Direction Following Single-Eye Blinks

**Gerrit Maus^1^ and Therese Collins^2^**

^1^Nanyang Technological University, Singapore; ^2^Universite Paris Descartes, France

When a target is displaced repeatedly during eye blinks, the oculomotor system adapts: The initial gaze direction after each eye blink becomes biased towards the new target position, without the subject noticing. Previously shown for repeated identical displacements, we now present evidence of such gaze adaptation after random target steps, and after just one blink. Observers were instructed to fixate a target dot. Eye blinks triggered random target steps left or right (between 0.1° and 1.0°), and observers reported the perceived direction of each step. The change in gaze direction from one blink to the next correlated with the retinal position of the target after the previous blink. This correlation was reduced when observers perceived the previous step accurately. This is evidence for a fast-acting adaptive mechanism that compensates for oculomotor noise when visual changes during blinks are not perceived as object motion.

## Brain Areas Associated With Getting Back on Track After Unexpected Events

**Andrew J. Anderson^1^, Matthew J. Stainer^1^, R. H. S. Carpenter^2^ and Peter Brotchie^3^**

^1^Department of Optometry & Vision Sciences, The University of Melbourne, Australia; ^2^Gonville and Caius College, University of Cambridge, Cambridge, UK; ^3^Clinical School, St. Vincent’s Hospital, Fitzroy, Australia

Unexpected events disrupt our routines, requiring us to suppress further habitual actions and engage conscious control to return us to our routines quickly. An oculomotor model (Anderson & Carpenter, 2010) involves an observer viewing a repeating sequence of targets (left-left-left-right). Occasionally an out-of-sequence target appears (an interruption) after which the sequence returns but at a randomized starting position. Here, we found that fMRI activity increased in the left insula cortex and left inferior temporal gyrus during interruptions. Subsequent appearance of the low-frequency target (right), which unambiguously determines the returned sequence’s phase and so gets the observer back on track, produced multiple areas of increased activity, including the inferior parietal lobe, left insula cortex, and right premotor cortex. We confirm the role of insular cortex during error processing and find a temporal sequence of activation we believe better captures, compared to simpler task-switching paradigms, how disruptions occur in the natural world.

## The Unique Contribution of Rate of Visual Information Processing to Cognition Across the Lifespan

**Deena Ebaid, Sheila Crewther, Kirsty MacCalman and Ben Ong**

La Trobe University, Melbourne Campus, Victoria, Australia

Cognitive decline across the lifespan is commonly documented in terms of motor reaction times though the unique contribution that rate of visual information processing (VIP) makes to such decline has seldom been explored. Thus, in the current study, cognitive reaction times (CRT) were measured using two tasks—Change Detection (CD) and Inspection Time (IT)—and an alphanumeric task of Rapid Automatic Naming (RAN) was used to explore rate of VIP in 70 young (18–30 years) and 40 older participants (38–78 years). CD was significantly slower in older compared to younger participants and although RAN was not significantly correlated with age, it correlated with threshold times for CD, suggesting that the rate of VIP effects CRT in older age groups. Rate of VIP as opposed to motor/verbal reaction times should be considered when assessing mental health and well-being of healthy older adults and clinical populations.

## Asymmetric Representation of Upper and Lower Visual Fields in Oculomotor Maps

**Zhiguo Wang^1^, Benchi Wang^2^ and Matthew Finkbeiner^1^**

^1^Department of Cognitive Science, Macquarie University, Australia; ^2^Department of Experimental and Applied Psychology, Vrije Universiteit Amsterdam, The Netherlands

The striate area devoted to the lower visual field (LVF) is larger than that devoted to the upper visual field (UVF). A similar anatomical asymmetry also exists in the LGN. Here, we show an opposite visual field asymmetry in oculomotor maps (e.g., the superior colliculus), taking advantage of two experimental tasks that are known to modulate the direction and amplitude of saccades. The participants made visually guided saccades. In Experiment 1, the saccade target was accompanied by a visual distractor. The distractor biased the direction of saccades away and this bias was much stronger for LVF targets. In Experiment 2, the fixation stimulus was removed shortly before the presentation of the saccade target. This temporal gap reduced the amplitude of saccades and this reduction was stronger for saccades towards UVF targets. These results show that the representation of meridians and eccentricities in the LVF are both compressed in oculomotor maps.

## Eyes, Faces, and Reading

## The Proportion of Eye Movements Associated With Enumeration Error Checking Does Not Increase With Set Size in Grouped Displays

**Jason Forte**

The University of Melbourne, Australia

The time taken to enumerate a number of dots increases with set size and is accompanied by increased eye movements. Some of these eye movements may occur because of a greater need for error checking as spatial complexity increases in randomly grouped displays. We estimated the ratio of error checking eye movements in 35 undergraduates by grouping dots at 4 distinct locations for set sizes 1 to 16 and determining how often previously fixated dot locations were revisited during enumeration. Enumeration was faster with fewer saccades when the four locations of dots had equal number. While the number of eye movements to previously fixated locations generally increased with set size, the proportion of potentially error checking saccades was not related to set size. Our results suggest that error checking during enumeration of random dot displays may depend on spatial complexity but that error checking occurs at a constant rate of numerical processing

## Position Dependency of the Slow Pupil Constriction to Intense Blue Light

**Koji Tanaka^1^ and Kowa Koida^2^**

^1^Department of Computer Science and Engineering, Toyohashi University of Technology, Japan; ^2^Electronics-Inspired Interdisciplinary Research Institute, Toyohashi University of Technology, Japan

We measured pupillary light responses, which are a representative non-image forming function of the visual system, and examined the effect of stimulus intensity, size, and color. By using a high luminance and large display, we successfully demonstrated that the intense blue light stimulus (450 nm, 12.9 lux) evoked a slow continuous constriction of the pupil for both macaque and human observers. The slow constriction was particularly prominent when the stimulus was displayed at the central visual field, and was not apparent in the far peripheral visual field (>12°) even if the amplitude of the constriction was matched.

These results indicate that current display is capable to drive slow and irradiance coding photoreceptors, putative intrinsically photosensitive retinal ganglion cell, and suggest that they are sensitive at the central visual field. Further physiological study of non-image forming nuclei in the brain is necessary.

## Relation of Rapid Automatized Naming (RAN) and Eye Movements in Young Learner Readers

**Jessica Peters, Jessica Taylor, Daniel Crewther, Alana Cross and Sheila Crewther**

La Trobe University, Australia

RAN successfully differentiates good readers from poor/dyslexic readers and is an established predictor of reading ability. It has been interpreted to reflect phonological processing or conversely as the ability to coordinate sequential processing of multiple visual stimuli. In an attempt to unravel which processes underpin RAN and thus determine how RAN is related to reading, eye tracking was used. Results indicate that typical readers not only had better RAN performances than poor/dyslexic readers, but demonstrated significantly shorter fixation duration’s and fewer fixations per stimulus (fixation efficiency). Hierarchical regression revealed eye movements (fixation duration and fixation efficiency) together predicted 79% to 99% of the variance for RAN, after controlling for phonological awareness. Overall, the findings suggest that RAN performance is much more closely related to eye movement function than phonological skill, and highlight that poor/dyslexic readers require longer duration of fixations to correctly identify and access the name of familiar objects.

## Activating Spatial Frequency Channels Is Sufficient to Benefit the Perception of a Fearful Face

**Li-Chuan Hsu^1^, Chia-Yao Lin^2^ and Yi-Min Tien^3^**

^1^School of Medicine, China Medical University, Taiwan; ^2^Graduate Institute of Neural and Cognitive Sciences, China Medical University, Taiwan; ^3^Department of Psychology, Chung Shan Medical University, Taiwan

Primary vision segregates information by distinct spatial frequencies (SF). Low-spatial frequencies (LSF) carry coarse information whereas high-spatial frequencies (HSF) carry fine details. Spatial frequency-specific processing also contributes to emotion processing, especially the LSF for fearful faces. We aimed to investigate how SF information modulates fearful face perception by using the priming paradigm. In Experiment 1, we adopt a priming paradigm in which target faces with two expressions (fearful or happy) were primed with face from the same emotional category (fearful-fearful or happy-happy) or Gabor. All presented primes were filtered and have five kinds of SF content. Results revealed clear emotional repetition priming effects for both fearful and happy faces within LSF and HSF respectively. Gabor produced clear priming effect only for fearful face, while failed to prime happy face at all SF conditions. This implies that a LSF-filtered Gabor alone is sufficient to facilitate perception of a fearful face.

## Does a Human-Like Appearance Increase Familiarity?

**Young Hun Sun and Woo Hyun Jung**

Department of Psychology, Chungbuk National University, South Korea

As technology advances, artificial agents such as dolls, robots, or animation characters become human look-alike. This study examined whether human-like appearance increases positive feeling towards the images of diverse images of artificial agents. For the purpose of ascertaining the relationship between the positive and negative feelings, we measured both familiarity and gruesomeness as the representative. Participants were asked to rate the degree of human-likeness and familiarity/gruesomeness when a series of pictures of artificial agents were presented to them via computer monitor. The result showed not a linear but a quadratic relationship in both responses of familiarity and gruesomeness. The middle range of human-like images evoked a lower familiarity and higher gruesomeness.

These findings suggest that the stimuli similar to human face may give rise to the uncanny valley effect (Mori, 1970).

## Anger or Disgust? Get Confused by Face Features!

**Yu-Pei Lin^1^, Yi-Min Tien^1^, Chia-Yao Lin^2^ and Li-Chuan Hsu^3^**

^1^Department of Psychology, Chung-Shan Medical University, Taiwan; ^2^Institute of Neural and Cognitive Sciences, China Medical University, China; ^3^Medical College, China Medical University, China

The degree of distinctiveness between emotions is not uniform. We aimed to investigate whether the confusion between different facial expressions manifests. In Experiment 1, participants were asked to judge emotional category and intensity of presented expression. Results revealed participants were more likely to confuse disgust with low intensity anger compared to other emotions. We adopted affective priming paradigm and manipulated prime types with different part of facial expressions presented in Experiments 2, 3A, and 3B: whole face, upper half, and lower half, respectively. We found angry prime would facilitate participants’ performance to judge the target face (Experiment 2). The angry upper faces would also enhance the performance of judging a disgusting face (Experiment 3A). No such priming effect was found in lower-half condition (Experiment 3B). Collectively, our findings suggest there was confusion between anger and low intensity disgust, and that this confusion resulted from overlapping the facial features on upper half faces.

## The Effect of Surround Emotion Faces on Facial Expression Identification

**Po-Shiuan Tsai and Pi-Chun Huang**

Department of Psychology, National Cheng-Kung University, Tainan, Taiwan

It has been shown by using a simple pattern stimulus that the discrimination/identification ability of a target is influenced by its surrounding stimulus. We investigated whether the surround effect also occurs in emotion face identification. We used emotion faces morphed at nine levels between anger and happy as the target, which was surrounded by four different types of emotion faces (angry, happy, neutral and inverted faces). Participants had to categorize the target facial expressions as happy or angry. We measured the response proportion of happy at 50% of the point of subjective equality (PSE) by using constant stimulus method. Results showed the PSE surrounded by happy faces to be lower than that surrounded by neutral faces. However, the angry and inverted surround faces caused the PSE unaffected. In conclusion, the target face looks happier when surrounded by happy faces but does not when surrounded with neutral, angry or inverted faces.

Acknowledgement: This work was supported by MOST 105-2420-H-006-001-MY2 (to P. C. H.).

## Seeing the Mood of a Crowd: Ensemble Expressions for a Group of Different Identities

**Markus F. Neumann^1^, Sarah Griffiths^2^, Romina Palermo^1^, Linda Jeffery^1^ and Gillian Rhodes^1^**

^1^ARC Centre of Excellence in Cognition and its Disorders, School of Psychology, The University of Western Australia, Australia; ^2^School of Experimental Psychology, University of Bristol, UK

Can we judge facial expressions accurately from faces that appear as part of a crowd? Previous work has demonstrated that people encode the mean facial expression when seeing “groups” that contain several exemplars of a single person’s face. Does ensemble coding of expression also occur for natural groups, in which group contains different peoples’ faces? Here, we provide evidence that participants extract the mean expression for such natural crowds, by showing that their judgements of the intensity of individuals’ facial expressions (anger or happiness) are biased towards the group mean expression intensity. These findings demonstrate that people can utilize ensemble coding to determine the mood of a naturalistic crowd at a glance. The bias towards the group mean expression could be adaptive for participants in order to reduce uncertainty about inaccurately encoded individual expressions, by integrating information about the accurate group average.

## The Timecourse of Expression Aftereffects

**Nichola Burton, Linda Jeffery, Jack Bonner and Gillian Rhodes**

ARC Centre of Excellence in Cognition and its Disorders, School of Psychology, The University of Western Australia, Australia

We investigated the timecourse of the expression aftereffect, an adaptation aftereffect that biases perception of facial expressions towards the opposite of the adapted expression. In Experiment 1, we examined the effect of the duration of adaptation and test stimuli on the size of the aftereffect. We found that the aftereffect builds up logarithmically and decays exponentially, a pattern also found for facial identity and figural face aftereffects, and for lower level visual aftereffects. This “classic” timecourse is consistent with a perceptual locus for expression aftereffects. We also found that significant aftereffects were still present as long as 3200 ms after adaptation. We extended our examination of the longevity of the aftereffect in Experiment 2 by inserting a stimulus-free gap between adaptation and test. A significant expression aftereffect was still present 32 s after adaptation. The persistence of the aftereffect suggests that this effect may have a considerable impact on day-to-day expression perception.

## The Presence of External Features Eliminates the Own-Race Bias

**David Keeble^1^, Hoo Keat Wong^1^ and Ian Stephen^2^**

^1^University of Nottingham Malaysia Campus, School of Psychology, Malaysia; ^2^Macquarie University, Australia

Recognition of unfamiliar faces relies heavily on external features (Henderson, Williams, & Falk, 2005; Sporer & Horry, 2011; but see Toseeb, Keeble, & Bryant, 2012). However, most studies on own-race bias (ORB) have focused on the recognition of internal features, and relatively little is known about to what extent external features affect the ORB. Here, in a yes/no face recognition task, the ORB was pronounced across ethnic groups in a multi-ethnic Asian population (Malaysian-Malay, Malaysian-Chinese, Malaysian-Indian, and Caucasian) when faces were presented with only internal features (Experiment 1), but this effect disappeared when faces were presented with external features (Experiment 2).

Further, the absence of external features was disruptive to recognition of other-race faces but not own-race faces. This suggests that different processing mechanisms may be involved for own- and other-race faces, with internal features of own-race faces being processed more efficiently while external features dominate representations of other-race faces.

## Does Ensemble Coding of Face Identity in Congenital Prosopagnosia Reflect Image-Based or Identity-Based Averaging?

**Matthew Robson, Romina Palermo, Linda Jeffery and Markus Neumann**

ARC Centre of Excellence in Cognition and its Disorders, School of Psychology, University of Western Australia, Australia

Individuals with congenital prosopagnosia (CP) have difficulties processing individual facial identity but their ability to extract average identity information for crowds of faces (ensemble coding) has been argued to be unimpaired. The present study asks whether apparently intact ensemble coding for face identity in prosopagnosia may reflect reliance on image-specific averaging rather than the abstraction of an average identity. In two tasks, 12 prosopagnosic and 20 control participants judged whether target faces were either the same image (identical photograph), or the same person (different photographs of the same person) as one of the faces comprising a previously presented set. Ensemble coding was assessed as the extent to which people endorsed identity averages of the whole group (using either group photographs, or new photographs of the same people) as seen members. Results are discussed with reference to current theories of CP and face-specific identity mechanisms.

## Faces Are Holistically Processed When Basic First-Order Configuration Is Maintained: The Case of Yaw and Pitch

**Simone Favelle^1^ and Rachel Robbins^2^**

^1^University of Wollongong, Australia; ^2^Western Sydney University, Australia

The composite effect is a widely recognised marker of holistic processing in upright face perception. Typically, these effects are examined for front views of faces. More recently, studies have investigated whether this marker is evident for faces viewed in a wider variety of conditions including motion and changes in view. Here, we use a composite task with top-bottom and left-right split composites to show that in addition to front and yaw rotated views of faces (also see McKone, 2008), holistic processing does occur for some, but not all, views of faces rotated in pitch. The results suggest that only views of faces that can be matched to a basic first-order representation of a face, engage holistic processing mechanisms.

## Is There an Other-Race Effect for Detection of Gaze Direction?

**Jemma Collova, Nadine Kloth, Kate Crookes, Gillian Rhodes and Nichola Burton**

ARC Centre of Excellence in Cognition and its Disorders, School of Psychology, University of Western Australia, Australia

Other-race effects in face perception have been widely studied due to their theoretical and social importance. However, no previous studies have investigated whether an other-race effect exists for the perception of gaze direction. Here, Asian and Caucasian participants classified the gaze direction (direct or averted) of own- and other-race faces in two experiments. No evidence was found for an other-race effect in gaze direction perception. Participants did show an other-race effect on established tests of mental-state attribution and identity recognition suggesting no lack of own-race expertise. Participants were also less accurate at perceiving gaze direction in inverted than upright faces, suggesting the task did tap face processing mechanisms. These findings argue that gaze direction is a cue that remains unaffected by the race of the face. This is a plausible conclusion given the evolutionary importance of detecting gaze direction and the similarity of this visual cue across different race faces.

## Perceptual Expertise for Other-Race Faces: So You Think They All Look Alike?

**Bianca Thorup and Paul Chang**

School of Psychology and Social Science, Edith Cowan University, Australia

There is an adage that “all Asians look alike.” The perceptual expertise hypothesis proposes that differential processing of own-race versus other-race faces leads to a bias towards recognising own-race faces, presumably resulting from finer processing and an expertise effect. This study extended previous research on the own-race bias to a categorisation task to examine whether perceptual expertise for own-race faces can be transferred to higher performance at categorising own-race faces by their national origin. It was hypothesised that Asian participants would demonstrate higher accuracy and confidence at categorising Asian face stimuli compared to Caucasian participants. Asian participants performed significantly above chance in correctly identifying the national origin of Asian faces, and were significantly more accurate and confident than Caucasian participants, who performed at chance level. The results suggest that Asian faces do not all look alike, and through experience and expertise, participants categorise Asian faces at a high level.

## Extensions and Constraints on Interaction Between Face and Expert Object Recognition: A Behavioral Study on Bird Expertise

**Chun-Chia Kung and Chiu-Yuen Chen**

Department of Psychology, National Cheng Kung University, Tainan, Taiwan

Although studies on human face recognition abound, its interaction with expert-object processing remains less addressed. The extant evidence exist mostly with car experts and under some temporally demanding task. To extend beyond specific expert domain and to further explore its constraints, the current study tested 18 bird experts and 18 novices with multimodal bird expertise measures (Experiment 1), composite face and bird task (Experiment 2), and the alternative two-back face-bird interference task (Experiment 3). Most interestingly (Experiment 3), we not only replicated the face-bird (previously face/car) interactions (that the higher the bird expertise, the higher the interference on faces), but also found the other end of the same interaction namely that the higher holistic processing for faces, the larger interference on birds! Together, these results comply with (that not all experts process objects of expertise with face-like representations, Experiment 2) and extend previous studies, and further strengthen the claim that faces and expert object processing share highly overlapping resources.

## Is Visual Problem Solving Dependent on Vocabulary Abilities in Individuals With Intellectual Disability?

**Chantanee Mungkhetklang, Sheila Crewther, Edith Bavin and Nahal Goharpey**

Latrobe University, Australia

Visual problem solving generally requires ability to perceive and an attempt to understand what has been seen in order to generate and evaluate a solution. The possible influence of language on visual problem solving has been unclear. Thus, it is important to consider the unique contributions of vocabulary abilities when investigating variation on nonverbal test results. The study compared individuals with Intellectual Disability and children with Typical Development of comparable mental age in order to determine the amount of variance contributed by expressive and receptive vocabulary scores on nonverbal scores. There were no significant differences in nonverbal scores and receptive vocabulary scores but in expressive vocabulary scores between two groups. Although visual problem solving as measuring by nonverbal ability assessments thought they are not related to any language components, our finding suggested that both expressive and receptive vocabulary ability plays a role in visual problem solving.

## The Role of Efficiency in the Relationship Between Rapid Automatised Naming (RAN), Orthographic Knowledge, and Reading

**Kamariani Houlis, Troy Visser, John Hogben, Jeneva Ohan, Mike Anderson and Steven Heath**

University of Western Australia, Australia

There has been confusion in the literature regarding the contribution of orthographic knowledge to the RAN/reading relationship due to the multiple facets of orthographic knowledge and tasks used to measure this construct. Additionally, until now the role of efficiency in this relationship has been neglected. This study tested 179 Year 6 children and sought to elucidate the role of efficiency in the relationships between RAN, orthographic knowledge and reading by examining: (a) both types of RAN, (b) accuracy, latency, and efficiency of experimental orthographic knowledge measures (mental graphemic representations and generic orthographic knowledge), (c) both untimed and speeded reading. Results suggest that the relationship between RAN and reading is partially mediated through efficiency of mental graphemic representations. However, generic orthographic knowledge had a minor effect on speeded reading only. Furthermore, the contribution of the RAN types overlapped in their relationships with speeded reading and efficiency of mental graphemic representations.

## Visual Search on Small Display Area by Scrolling or Moving Windows

**Yumiko Fujii^1^, Saho Ayabe-Kanamura^2^ and Hiromi Morita^1^**

^1^Graduate School of Library, Information and Media Studies, University of Tsukuba, Japan; ^2^Faculty of Human Sciences, University of Tsukuba, Japan

When we look at images within the limited display area of smartphones, we are forced to see under a condition in which the peripheral visual field is unavailable and visible parts of the images are overwritten sequentially. We investigated these conditions for further knowledge on visual perception and perception with smartphones.

Experiments: Participants searched for targets serially through a window smaller than the search array under Image-Scroll Condition in which they scrolled images in a fixed window and under Window-Move Condition in which they moved the window to see a fixed image.

Results: The search time was longer for Image-Scroll Condition than for Window-Move Condition. The rate of increase in search time was lower for Image-Scroll Condition.

Discussion: The manner of scrolling affected visual search performance. The analysis of scanning path showed that we scan images differently depending on whether we move the image or the window.

## Are Vertical Arrangements More Aesthetic Than Horizontal Arrangements for Chinese Characters?

**Lin Shi and Yue Zhang**

Kunming University of Science and Technology, China

Chinese characters were used to vertical arrangements in traditional calligraphy works and publications while the horizontal arrangements dominated at present. We were interested in the visual aesthetics of vertical and horizontal arrangements of Chinese characters.

Twenty participants were asked to make aesthetic arrangements of the Chinese character images in the horizontal or vertical direction using drag-and-drop operations controlled by the mouse. An arrangement consisted of seven Chinese character images, which were arranged initially at random positions near the central vertical or horizontal line on a gray square background. Thirty vertical arrangements and thirty horizontal arrangements were included alternately in each participant’s experiment. After the arrangements of all participants, evaluations were given by them with values ranging from 1 to 10, and high values indicated better visual aesthetic of arrangements. The participants’ eye movements and operations were both recorded in the experiment. Results showed that (a) the duration of vertical arrangement operations was lower than that of horizontal arrangement operations and (b) the evaluation values of vertical arrangements were higher than those of horizontal arrangements. The results indicated that vertical arrangements tended to be more visually aesthetic than horizontal arrangements for Chinese characters.

